# Antibiofilm and Antivirulence Activities of Gold and Zinc Oxide Nanoparticles Synthesized from Kimchi-Isolated *Leuconostoc* sp. Strain C2

**DOI:** 10.3390/antibiotics11111524

**Published:** 2022-11-01

**Authors:** Min-Gyun Kang, Fazlurrahman Khan, Du-Min Jo, DoKyung Oh, Nazia Tabassum, Young-Mog Kim

**Affiliations:** 1 Department of Food Science and Technology, Pukyong National University, Busan 48513, Korea; 2 Marine Integrated Biomedical Technology Center, The National Key Research Institutes in Universities, Pukyong National University, Busan 48513, Korea; 3 Research Center for Marine Integrated Bionics Technology, Pukyong National University, Busan 48513, Korea

**Keywords:** *Leuconostoc* sp. strain C2, supernatant, nanoparticles, *Pseudomonas aeruginosa*, *Staphylococcus aureus*, antibiofilm, antivirulence

## Abstract

The rapid emergence of antimicrobial resistance (AMR) among bacterial pathogens results in antimicrobial treatment failure and the high mortality rate associated with AMR. The application of nanoparticles synthesized from probiotics will be widely accepted due to their efficacy and biocompatibility in treating microbial infections in humans. The current work sought to isolate and identify lactic acid bacteria (LAB) from Kimchi. Based on 16S rRNA gene sequencing, the LAB isolate C2 was identified as a member of the genus *Leuconostoc*. The obtained supernatant from *Leuconostoc* sp. strain C2 was employed for the green synthesis of metal (AuNPs) and metal oxide (ZnONPs) nanoparticles. UV–vis absorption spectra, FTIR analysis, XRD, DLS, FE-TEM, and EDS mapping were used to fully characterize these C2-AuNPs and C2-ZnONPs. The C2-AuNPs were found to be spherical in shape, with a size of 47.77 ± 5.7 nm and zeta potential of −19.35 ± 0.67 mV. The C2-ZnONPs were observed to be rod-shaped and 173.77 ± 14.53 nm in size. The C2-ZnONPs zeta potential was determined to be 26.62 ± 0.35 mV. The C2-AuNPs and C2-ZnONPs were shown to have antimicrobial activity against different pathogens. Furthermore, these nanoparticles inhibited the growth of *Candida albicans*. The antibiofilm and antivirulence properties of these NPs against *Pseudomonas aeruginosa* and *Staphylococcus aureus* were thoroughly investigated. C2-AuNPs were reported to be antibiofilm and antivirulence against *P. aeruginosa*, whereas C2-ZnONPs were antibiofilm and antivirulence against both *P. aeruginosa* and *S. aureus*. Furthermore, these nanoparticles disrupted the preformed mature biofilm of *P. aeruginosa* and *S. aureus*. The inhibitory impact was discovered to be concentration-dependent. The current research demonstrated that C2-AuNPs and C2-ZnONPs exhibited potential inhibitory effects on the biofilm and virulence features of bacterial pathogens. Further studies are needed to unravel the molecular mechanism behind biofilm inhibition and virulence attenuation.

## 1. Introduction

The emergence of antimicrobial resistance (AMR) among bacterial pathogens has become a major concern globally [1]. The problem arises due to bacterial resistance, imposes a significant economic burden, and necessitates large sums of money to treat the patients [2]. According to a recent study, around 4.95 million individuals died as a result of bacterial AMR [3]. The ESKAPE (*Enterococcus faecium*, *Staphylococcus aureus*, *Klebsiella pneumoniae*, *Acinetobacter baumannii*, *Pseudomonas aeruginosa*, and *Enterobacter* spp.) are the top priority bacterial pathogens on the WHO’s comprehensive list of which new antimicrobial agents must be identified urgently [1,4]. Pathogens have several antimicrobial resistance mechanisms, including drug target alteration, cell permeability changes, increased efflux pump, genetic mutations, and the acquisition of mobile genetic elements [5,6]. Furthermore, these pathogens exhibit an adaptive mechanism in the form of biofilm development, which is a collection of microbial populations covered by self-produced polymeric substances [7]. The biofilms serve as a physical barrier against antimicrobial agents [8]. Biofilms also cause the formation of a small population of persister cells, which are the primary source of persistent and recurring infection [9]. Biofilm formation has been seen in human tissues such as skin, teeth, trachea, and urinary system surfaces, as well as the surfaces of various medical devices [10]. With the growing concern over bacterial pathogen infection and resistance, there is always a high demand for new drugs to tackle these diseases. Much progress has been made in treating infectious and non-infectious disorders due to the advancement of nanotechnology [11]. The use of nanoparticles has grown due to various benefits, including a large surface area, small size, stability, and biocompatibility [12]. Furthermore, the green technique for nanoparticle synthesis offers significant benefits over the chemical-based technique [13]. Green synthesis NPs have several benefits over chemical and physical approaches, including being easier to synthesize, eco-friendly, cost-effective, minimizing the release of harmful compounds into the environment, and producing stable and biocompatible NPs [14,15]. There are more and more reports on the production of nanoparticles from biologically derived materials such as plants, algae, fungi, bacteria, and animals [13]. Since probiotics, particularly lactic acid bacteria, have health benefits, they provide a number of benefits to human health and well-being. Several studies have found that LAB in the gut microbiota utilize a competitive exclusion strategy to avoid the colonization of pathogenic bacteria [16]. As a result, using LAB in nanoparticle synthesis would be another viable technique with highly biocompatible applicability for treating infections caused by bacterial pathogens. The current study is divided into three sections: (1) the isolation and identification of LAB from Kimchi; (2) the synthesis of metal (e.g., gold) and metal oxide (e.g., zinc oxide) nanoparticles using the LAB’s cell-free supernatant; and (3) the application of these nanoparticles to inhibit the biofilm formation and virulence properties of *S. aureus* and *P. aeruginosa* bacterial pathogens.

## 2. Results

### 2.1. Isolation and Identification of the Lactic Acid Bacteria

**Figure 1:**
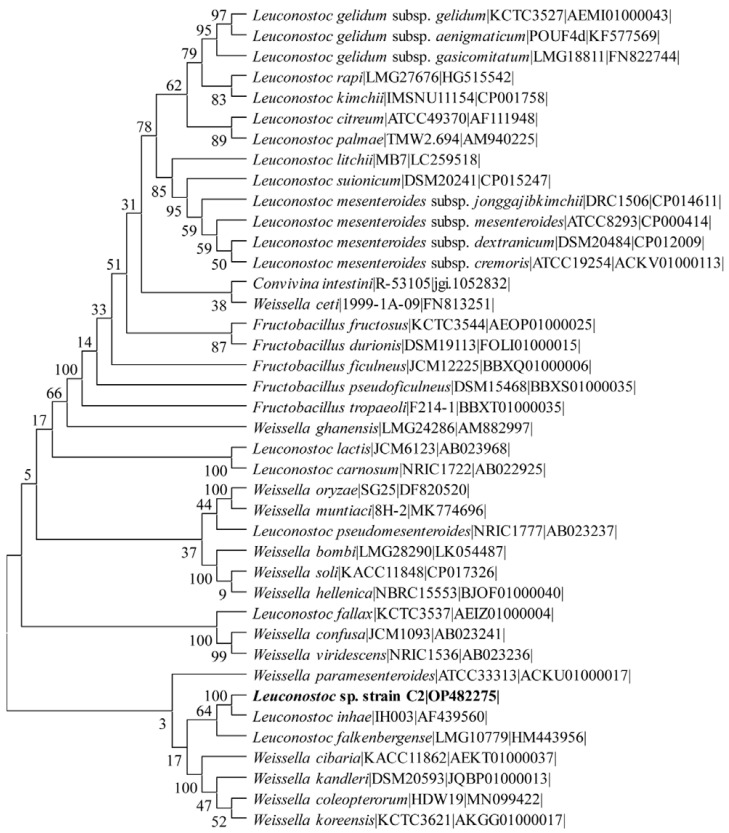
Phylogenetic tree of strain C2 based on the 16S rRNA gene sequence. The tree shows relations with the other LAB-type strain. The neighbor-joining tree was constructed using the bootstrap value of 1000 under the MEGA 11 package [17].

A total of 15 LAB strains were isolated from the fermented food product Kimchi from the Korean island of Jeju. The isolates were screened for antioxidant activity, and the best strain was chosen for the metal and metal–oxide nanoparticle synthesis. The isolate C2 was identified using 16S rRNA gene sequencing. The 16S rRNA gene sequence of strain C2 was determined to be most similar to the genus *Leuconostoc*. A blast search also reveals that it closely resembles several LAB strains from the genus *Weissella*. The phylogenetic tree analysis also reveals the presence of strain C2 between the branches of *Weissella paramesenteroides* and *Leuconostoc inhae* (Figure 1). We identified it as *Leuconostoc* sp. strain C2 based on the highest similarity with several LAB strains of the genus *Leconostoc*. The 16S rRNA gene sequence was submitted to GenBank under accession number OP482275.

### 2.2. Biosynthesis and Characterization of the Gold and Zinc Oxide Nanoparticles

**Figure 2:**
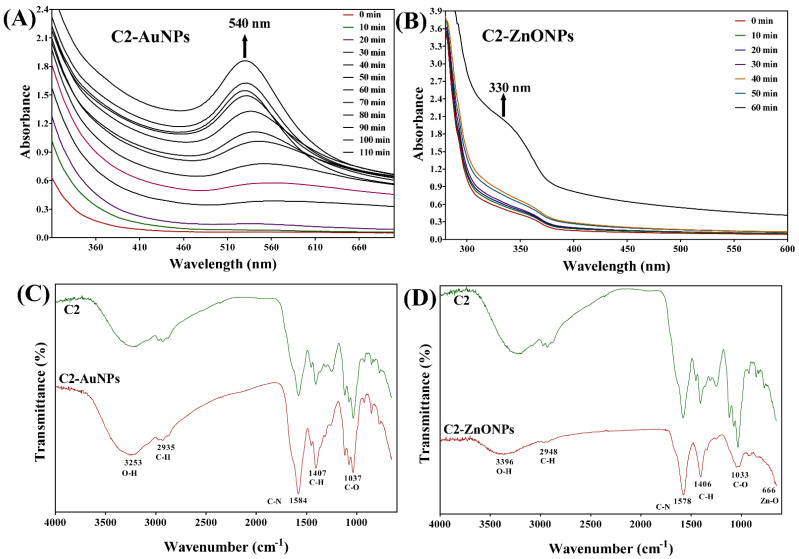
Spectroscopic analysis of the synthesized nanoparticles. (**A**) Absorption spectra of C2-AuNPs; (**B**) Absorption spectra of C2-ZnONPs; (**C**) FTIR spectra of the C2-supernatant and C2-AuNPs; and (**D**) FTIR spectra of C2-supernatant and C2-ZnONPs.

The supernatant of the LAB strain C2 was used as a reducing biological material for the synthesis of both metal and metal–oxide nanoparticles in a green synthesis strategy. The formation of AuNPs is indicated by the visible appearance of the final red-wine color from the initial yellow color of the reaction. In contrast, a white precipitated formation suggests the formation of ZnONPs. The manifestation of nanoparticles was further examined under the UV–vis absorption spectra, which show that particular specific absorption peaks increase with the reaction time. The absorption peak at 540 nm was found to constantly grow with the reaction in the case of AuNPs (Figure 2A), whereas the absorption peak at 330 nm was observed to increase over the course of the reaction in the case of ZnONPs (Figure 2B). Furthermore, the FTIR analysis of these nanoparticles was performed to record some of the distinctive vibration band that is particular to the nanoparticles (Figure 2C,D). The typical absorption peaks in the FTIR of C2-AuNPs are seen at 3253, 2935, 1584, 1407, and 1037 cm^−1^ (Figure 2C). Similar bands of absorption have previously been discovered in the FTIR, indicating their uniqueness to AuNPs [18]. Similarly, there are certain distinctive bands in the FTIR of C2-ZnONPs at 3396, 2948, 1578, 1406, and 1033 cm^−1^ (Figure 2D). Similar absorption bands have been observed in ZnONPs synthesized from *Marinobacter* sp. 2C8 and *Vibrio* sp. VLA [19]. The stretching of hydroxyl bonds is recognized as having an absorption peak at 3396 cm^−1^, a peak at 2948 cm^−1^ for the C-H, a peak at 1578 cm^−1^ for the C-N, a peak at 1406 cm^−1^ for the C-H, and a peak at 1033 cm^−1^ for the C-O stretching of amide II.

**Figure 3:**
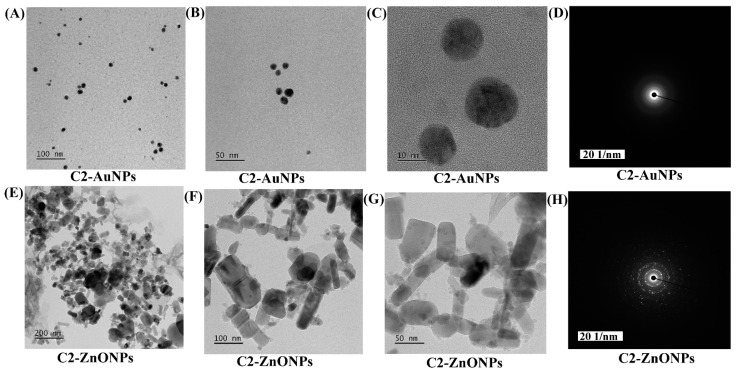
The morphology of nanoparticles was examined using FE-TEM. (**A**) FE-TEM image of C2-AuNPs at 100 nm resolution; (**B**) FE-TEM image of C2-AuNPs at 50 nm resolution; (**C**) FE-TEM image of C2-AuNPs at 10 nm resolution; (**D**) SAED of C2-AuNPs; (**E**) FE-TEM image of C2-ZnONPs at 200 nm resolution; (**F**) FE-TEM image of C2-ZnONPs at 100 nm resolution; (**G**) FE-TEM image of C2-ZnONPs at 50 nm resolution; and (**H**) SAED of C2-ZnONPs.

**Figure 4:**
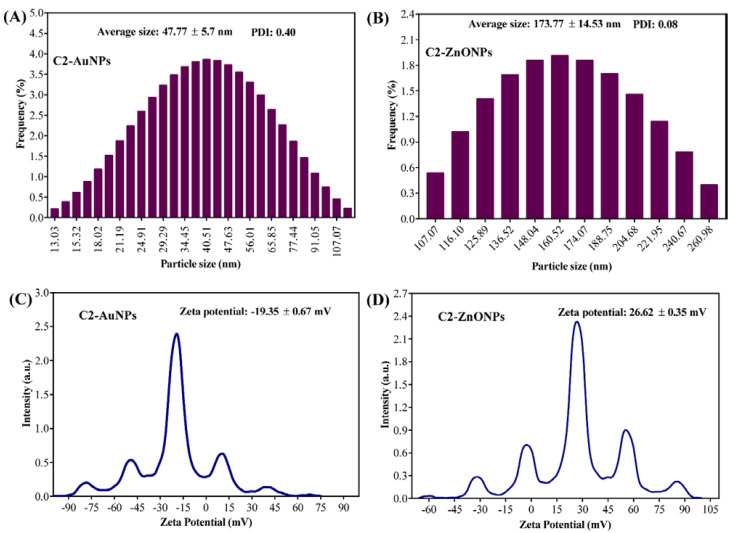
(**A**) DLS of C2-AuNPs; (**B**) DLS of C2-ZnONPs; (**C**) Zeta potential of C2-AuNPs; and (**D**) zeta potential of C2-ZnONPs.

The morphology of C2-AuNPs and C2-ZnONPs was examined with the assistance of field-emission transmission electron microscopy (FE-TEM) (Figure 3). The C2-AuNPs have a spherical shape, as seen in images acquired by FE-TEM with resolutions of 100, 50, and 10 nm (Figure 3A–C). C2-ZnONPs were discovered to be rod-shaped, as seen in FE-TEM images with resolutions of 200, 100, and 50 nm (Figure 3E–G). The size of C2-AuNPs was calculated by dynamic light scattering (DLS) to be 47.77 ± 5.7 nm, with a polydispersity index (PDI) of 0.40 (Figure 4A).

**Figure 5:**
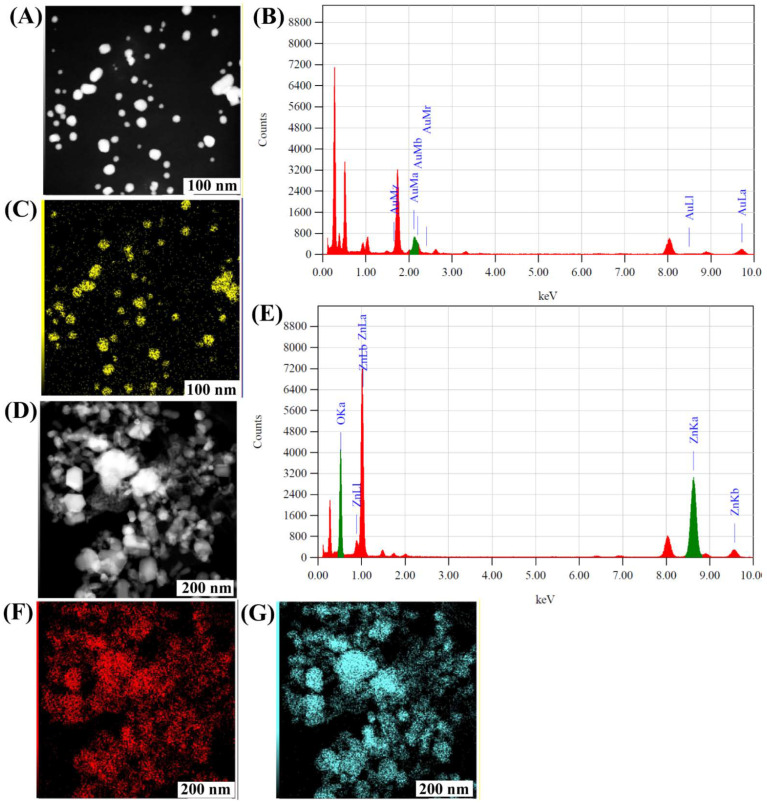
Energy-dispersive X-ray diffraction spectrum and EDS mapping of nanoparticles. (**A**) SEM elemental mapping of C2-AuNPs; (**B**) EDS spectrum of C2-AuNPs; (**C**) Au elemental map from C2-AuNPs; (**D**) SEM elemental mapping of C2-ZnONPs; (**E**) EDS spectrum of C2-ZnONPs; (**F**) O elemental map from C2-ZnONPs; and (**G**) Zn elemental map from C2-ZnONPs.

**Figure 6:**
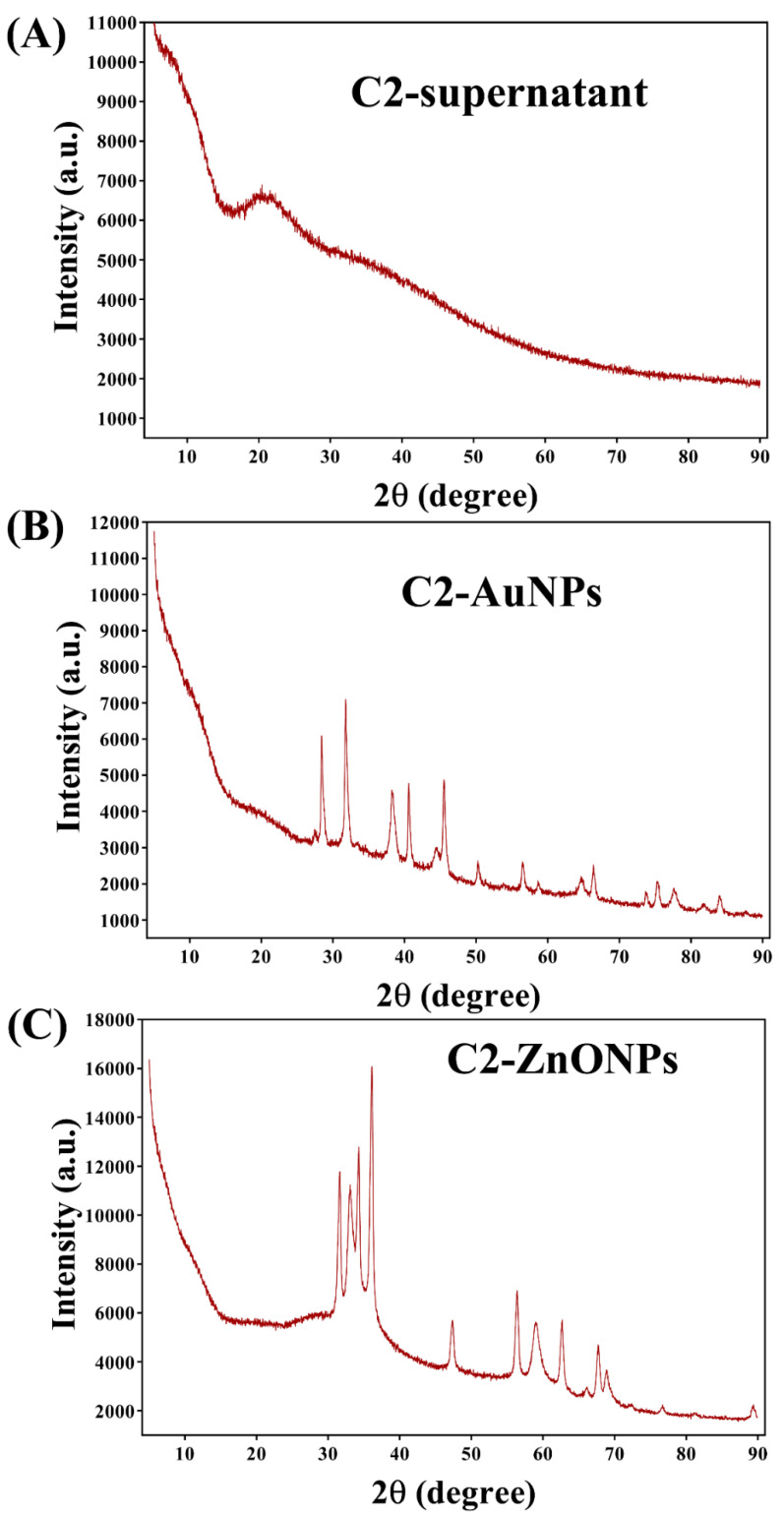
X-ray diffraction (XRD) spectra of nanoparticles. (**A**) Spectra of C2-supernatant; (**B**) Spectra of C2-AuNPs; and (**C**) Spectra of C2-ZnONPs.

Similarly, the size distribution of C2-ZnONPs determined by DLS was found to be with an average value of 173.77 ± 14.53 nm and PDI value of 0.08 (Figure 4B). The overall net charge on the surface of C2-AuNPs was found to be −19.35 ± 0.67 mV, while the net charge on the surface of C2-ZnONPs was 26.62 ± 0.35 mV (Figure 4C,D). EDS analysis was used to determine the elemental composition and mapping in C2-AuNPs and C2-ZnONPs (Figure 5). The EDS spectra and elemental mapping findings showed the presence of Au in C2-AuNPs (Figure 5B,C), as well as Zn and O in C2-ZnONPs (Figure 5E–G). XRD analysis was used to assess the crystallinity of these nanoparticles (Figure 6). Although no identifiable peaks were identified in the case of the C2-supernatant, a wide peak between 16 and 25° was observed (Figure 6A). The C2-AuNP diffraction peaks were seen at *2θ* values of 28.40, 31.76, 38.22, 40.59, 44.58, 50.28, 56.53, 64.75, 66.35, 75.25, 77.61, and 84.68 (Figure 6B). Similarly, the C2-ZnONP diffraction peaks were detected at *2θ* values of 34.28, 36.10, 47.36, 56.40, 58.97, 62.70, 67.69, 68.6, and 76.6 (Figure 6C).

### 2.3. Antimicrobial Effect of LAB Supernatant and Synthesized Nanoparticles

The growth inhibitory effects of strain C2 and the synthesized nanoparticles (C2-AuNPs and C2-ZnONPs) against different bacterial species, including the fungal pathogen *Candida albicans*, were investigated utilizing microbroth dilution methods. The antimicrobial results obtained in this study are reported in Table 1. The antimicrobial activity of the C2 supernatant against *S. aureus, K. pneumoniae, S. mutans, E. coli,* and *L. monocytogenes* was effective (>90% cell growth inhibition) at a 1/4-dilution of the prepared supernatant stock. Whereas *C. albicans* was found to be effective at a 1/2-dilution, *V. vulnificus* was effective at a 1/8-dilution of the supernatant.

The minimum inhibitory concentration (MIC) values of C2-AuNPs and C2-ZnONPs were discovered to be variable against these microbial pathogens. The MIC values of C2-AuNPs against Gram-negative bacteria such as *P. aeruginosa, L. monocytogenes*, and *E. coli* were determined to be >2048, 2048, and 1024 µg/mL, respectively. In contrast, the MIC values of C2-AuNPs against Gram-positive bacteria such as *S. aureus*, *S. mutans*, and *K. pneumonaie* were determined to be 1024, 512, and 512 µg/mL, respectively. Similarly, the MIC values of C2-ZnONPs against *P. aeruginosa* were >2048 µg/mL, whereas the MIC values against *S. aureus* were (512 µg/mL), which is four times lower than *P. aeruginosa*. The current investigation found that the MIC values of C2-AuNPs and C2-ZnONPs against Gram-negative bacteria were 2- and 4-fold higher, respectively, than for Gram-positive bacteria.

### 2.4. Antibiofilm Properties of the Nanoparticles toward Bacterial Pathogens

**Figure 7:**
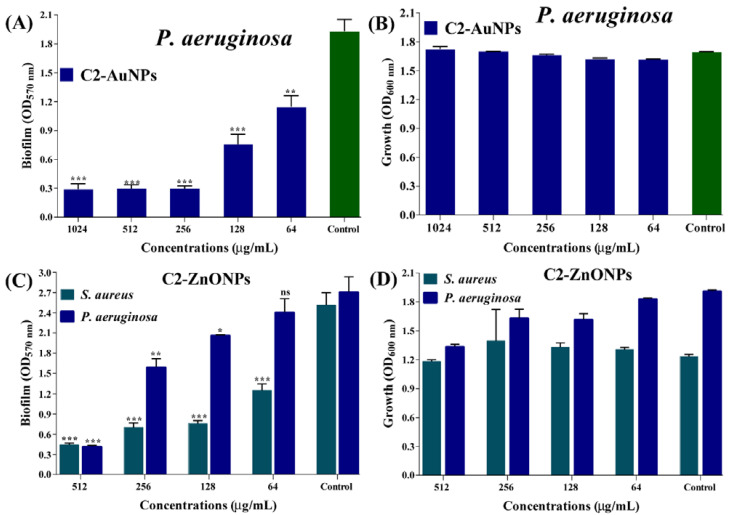
Biofilm inhibitory effect of nanoparticles towards *P. aeruginosa* and *S. aureus*. (**A**) Inhibition of *P. aeruginosa* biofilms by C2-AuNPs; (**B**) Cell growth of *P. aeruginosa* incubated with C2-AuNPs; (**C**) Inhibition of *P. aeruginosa* and *S. aureus* biofilms by C2-ZnONPs; and (**D**) Cell growth of *P. aeruginosa* and *S. aureus* incubated with C2-ZnONPs. The experiments were carried out using the crystal violet staining assays followed by measuring OD_570_, whereas the cell growth of bacteria incubated with nanoparticles was estimated at OD_600_. The statistical significance values ***, **, and * denote significance at *p* < 0.0001, *p* < 0.01, and *p* < 0.05—whereas ns denotes non-significance.

Crystal violet assays were used to assess the inhibitory impact of C2-AuNPs and C2-ZnONPs on *P. aeruginosa* and *S. aureus* biofilm formation at the early stage. The biofilm inhibition effect was achieved using sub-MIC values of C2-AuNPs and C2-ZnONPs. C2-AuNPs inhibited the biofilm formation solely against *P. aeruginosa*, whereas C2-ZnONPs inhibited biofilm formation against *P. aeruginosa* and *S. aureus*, respectively (Figure 7). C2-AuNPs and C2-ZnONPs dose-dependently inhibited these pathogens (Figure 7A,C). The maximal biofilm inhibition by C2-AuNPs against *P. aeruginosa* was determined to be 84.62% at 256 µg/mL. At a concentration of 512 µg/mL, the inhibitory effect of C2-ZnONPs on *P. aeruginosa* and *S. aureus* biofilms was 84.69% and 82.42%, respectively.

The cell growth of planktonic and adherent cells was measured at OD_600 nm_ to confirm that 100% of the cells were involved in biofilm formation in the presence of sub-MIC levels of C2-AuNPs and C2-ZnONPs. The growth findings demonstrated that *P. aeruginosa* and *S. aureus* cells were unaffected by the various concentration ranges of C2-AuNPs and C2-ZnONPs (Figure 7B,D). The biofilm inhibitory effects of C2-AuNPs and C2-ZnONPs on the biofilms of *P. aeruginosa* and *S. aureus* were also validated by examining the NPs in treated and untreated cells using scanning electron microscopy (SEM).

**Figure 8:**
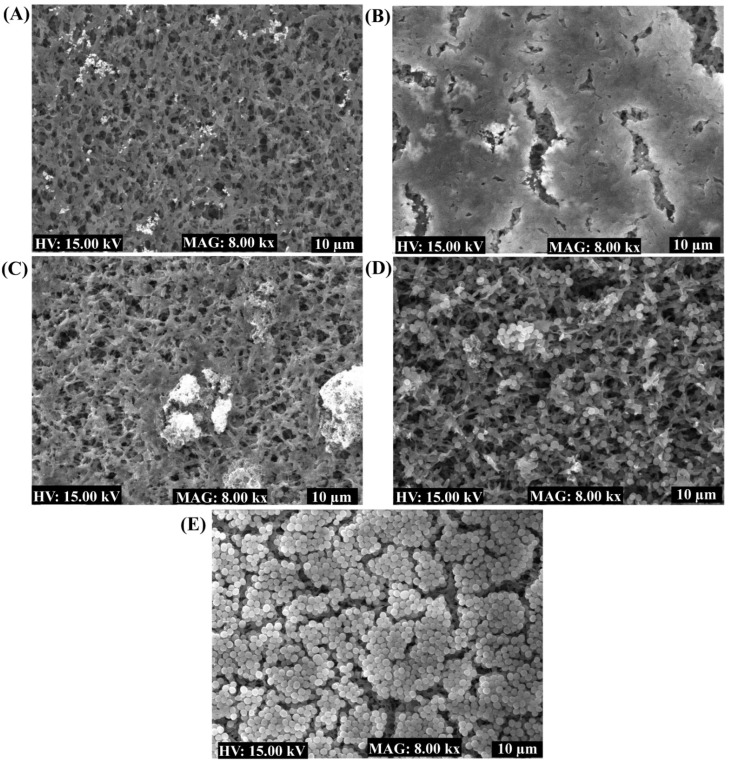
Imaging of biofilm cells of *P. aeruginosa* and *S. aureus* treated with C2-AuNPs and C2-ZnONPs using scanning electron microscopy. (**A**) Biofilm of *P. aeruginosa* treated with C2-AuNPs; (**B**) Biofilm of *P. aeruginosa* (untreated-control); (**C**) Biofilm of *P. aeruginosa* treated with C2-ZnONPs; (**D**) Biofilm of *S. aureus* treated with C2-ZnONPs; and (**E**) Biofilm of *S. aureus* (untreated-control).

The SEM image showed that *P. aeruginosa* cells treated with C2-AuNPs inhibit cell adhesion on the nylon membrane (Figure 8A), but untreated (control) cells displayed a thick biofilm cell adhering to the membrane (Figure 8B). Similarly, C2-ZnONPs was discovered to have an effect on the biofilm of *P. aeruginosa* and *S. aureus*. *P. aeruginosa* cells treated with C2-ZnONPs stopped adhering and ceased the biofilm formation on the surface of the nylon membrane (Figure 8C). In contrast, a few cells of *S. aureus* were observed to be attached in the presence of C2-ZnONPs (Figure 8D). Untreated (control) *S. aureus* cells were shown to produce highly thick biofilm architecture on the nylon membrane, similar to *P. aeruginosa*. The SEM imaging of cells treated with NPs demonstrated that these NPs inhibit biofilm formation.

### 2.5. Eradication of the Established Mature Biofilm

**Figure 9:**
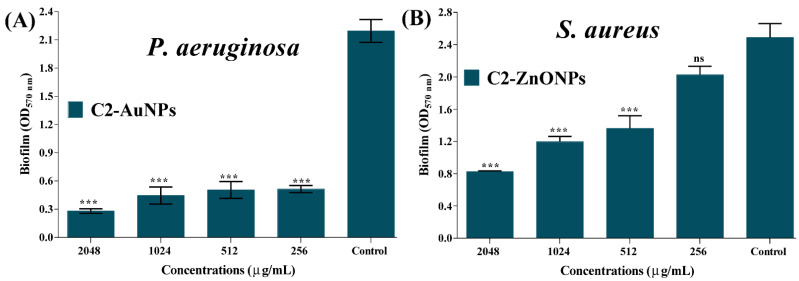
Analysis of the eradication of mature biofilm cells of *P. aeruginosa* and *S. aureus* by nanoparticles. (**A**) Eradication of the *P. aeruginosa* mature biofilm using C2-AuNPs and (**B**) eradication of *S. aureus* mature biofilm using C2-ZnONPs. Biofilm stained with crystal violet was used to determine eradication. The statistical significance *** denotes significance at *p* < 0.0001, and ns denotes non-significance.

The in vitro preformed-mature biofilms of *P. aeruginosa* and *S. aureus* were treated with C2-AuNPs and C2-ZnONPs at their above MIC, MIC, and sub-MIC concentrations. The eradication efficiency was assessed by quantifying the adherent cells using the crystal violet staining method. The results demonstrated that at the above MIC values, these NPs substantially eradicate the mature biofilm of *P. aeruginosa* and *S. aureus* (Figure 9A,B). The highest percentage of the eradication of mature *P. aeruginosa* biofilm by C2-AuNPs at a concentration of 2048 µg/mL was reported to be 87.75% (Figure 9A). Although C2-ZnONPs inhibit the biofilm formation in both *P. aeruginosa* and *S. aureus*, it is most effective in *S. aureus* mature biofilm. The maximal eradication of the mature *S. aureus* biofilm by C2-ZnONPs at a concentration of 2048 µg/mL was reported to be 66.8% (Figure 9B). Furthermore, the *S. aureus* mature biofilm eradication by C2-ZnONPs was concentration-dependent.

### 2.6. Inhibitory Effect of NPs on P. aeruginosa Motilities

**Figure 10:**
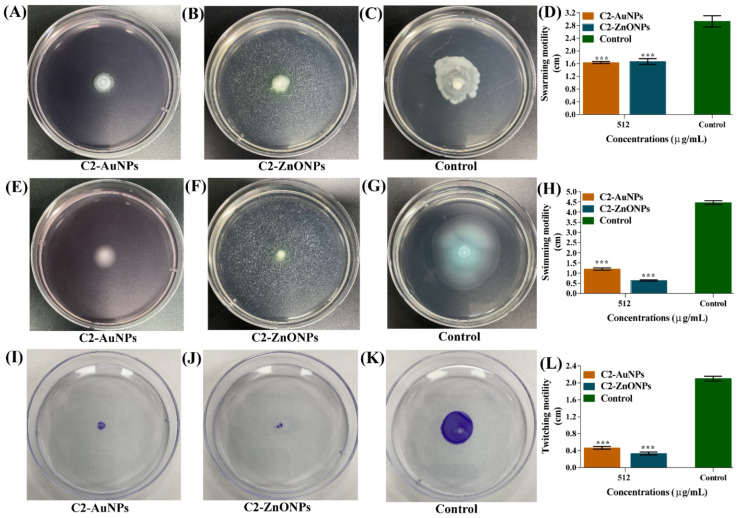
Inhibition of different types motilities of *P. aeruginosa*. (**A**) Swarming in the presence of C2-AuNPs; (**B**) Swarming in the presence of C2-ZnONPs; (**C**) Swarming of non-treated cells; (**D**) Bar graph showing swarming inhibitory values C2-AuNPs; (**E**) Swimming in the presence of C2-AuNPs; (**F**) Swimming in the presence of C2-ZnONPs; (**G**) Swimming of non-treated cells; (**H**) Bar graph showing swimming inhibitory values C2-AuNPs; (**I**) Twitching in the presence of C2-AuNPs; (**J**) Twitching in the presence of C2-ZnONPs; (**K**) Twitching of non-treated cells; and (**L**) Bar graph showing twitching inhibitory values C2-AuNPs. *** denotes significance at *p* < 0.0001.

Different motility patterns in *P. aeruginosa*, such as swimming, swarming, and twitching, play a significant role in biofilm development and pathogenicity. Inhibiting various forms of motility will be an essential step in reducing *P. aeruginosa* biofilm formation and virulence activities. The sub-MIC value of C2-AuNPs and C2-ZnONPs was utilized to assess the impairment of these motilities (Figure 10). C2-AuNPs (Figure 10A) and C2-ZnONPs (Figure 10B) both significantly inhibited the swarming motility on the agar plate surface when compared to the control (Figure 10C). Swarming inhibition by C2-AuNPs and C2-ZnONPs was determined to be 44.31% and 43.18%, respectively (Figure 10D).

Both C2-AuNPs and C2-ZnONPs significantly inhibited the swimming motility of *P. aeruginosa* at sub-MIC concentrations (Figure 10E,F), whereas the control cells displayed considerable swimming motility (Figure 10G). Swimming motility inhibition by C2-AuNPs and C2-ZnONPs was shown to be 73.13% and 85.82% effective, respectively, when compared to the control (Figure 10H). C2-AuNPs and C2-ZnONPs were similarly observed to drastically reduce twitching motility when compared to the controls (Figure 10I–K). The twitching motility inhibition by C2-AuNPs and C2-ZnONPs was calculated to be 77.77% and 84.12%, respectively (Figure 10L).

### 2.7. Virulence-Inhibitory Properties of the Nanoparticles

**Figure 11:**
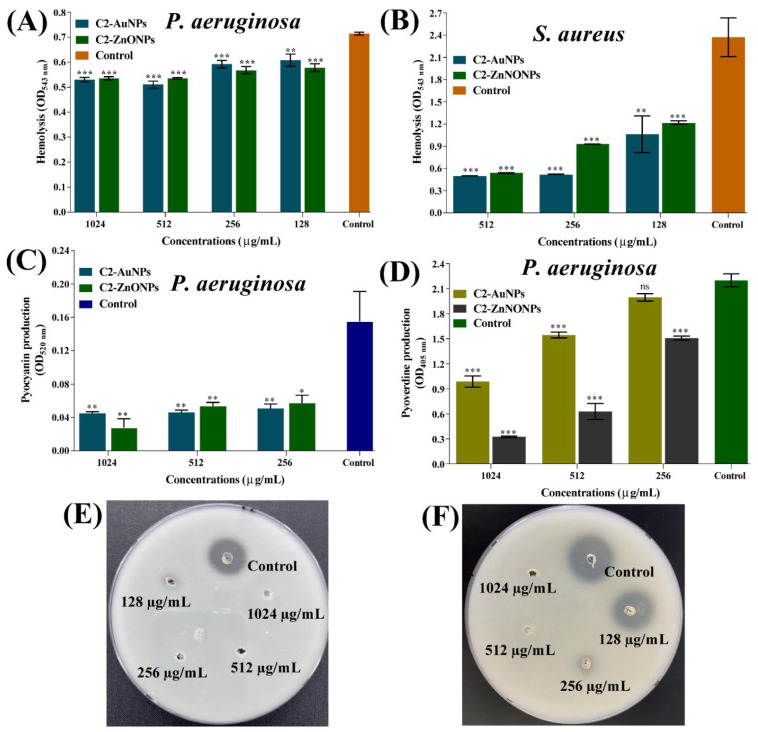
Inhibition of different virulence properties of *P. aeruginosa* and *S. aureus* by C2-AuNPs and C2-ZnONPs. (**A**) Inhibition of the hemolytic activity of *P. aeruginosa* by C2-AuNPs and C2-ZnONPs; (**B**) Inhibition of the hemolytic activity of *S. aureus* by C2-AuNPs and C2-ZnONPs, (**C**) Inhibition of the production of pyocyanin from *P. aeruginosa* by C2-AuNPs and C2-ZnONPs; (**D**) Inhibition of pyoverdine production from *P. aeruginosa* by C2-AuNPs and C2-ZnONPs; (**E**) Inhibition of protease activity of *P. aeruginosa* by C2-AuNPs; and (**F**) Inhibition of protease activity by C2-ZnONPs. ***, ** and * denote significance at *p* < 0.0001, *p* < 0.01, and *p* < 0.05.

A variety of virulence factors play a role in the pathogenesis of *P. aeruginosa* and *S. aureus* [20,21]. The effect of C2-AuNPs and C2-ZnONPs on *P. aeruginosa* pathogenicity characteristics such as hemolytic activity, pyocyanin, and pyoverdine production, and protease activity was studied at sub-MIC levels (Figure 11). In the case of *S. aureus*, we solely looked at the hemolytic effect of these NPs. In the case of *S. aureus*, however, we solely investigated the hemolytic effect of these NPs. However, there was no concentration-dependent inhibition of *P. aeruginosa* hemolytic activities by C2-AuNPs or C2-ZnONPs. However, at a concentration of 1024 µg/mL, C2-AuNPs and C2-ZnONPs inhibited the hemolytic activity by ~25% on *P. aeruginosa* (Figure 11A). The inhibition of *S. aureus* hemolytic activity by C2-AuNPs and C2-ZnONPs was highly significant and dose-dependent (Figure 11B). At a 512 µg/mL concentration, C2-AuNPs and C2-ZnONPs inhibited the hemolytic activity by 78.87% and 77.27%, respectively. C2-AuNPs and C2-ZnONPs were likewise discovered to dramatically inhibit pyocyanin virulence factor production from *P. aeruginosa* (Figure 11C). The inhibitory impact of C2-AuNPs and C2-ZnONPs on pyocyanin production was 71.07% and 82.15%, respectively, at 1024 µg/mL concentrations. Similarly, both C2-AuNPs and C2-ZnONPs inhibited the production of the *P. aeruginosa* pyoverdine virulence factor (Figure 11D). The inhibition was dose-dependent, with C2-AuNPs and C2-ZnONPs exhibiting a maximal inhibition of 55.1% and 85.24% at 1024 µg/mL, respectively.

In a casein agar plate assay, the *P. aeruginosa* protease activity was dramatically inhibited in the presence of C2-AuNPs and C2-ZnONPs at their sub-MIC value (Figure 11E,F). The supernatant produced from cells treated with C2-AuNPs and C2-ZnONPs at 1024 and 512 µg/mL concentrations had a negligible zone of casein protein digestion. The finding of these inhibitory effects by these NPs predicts that *P. aeruginosa* and *S. aureus* would be non-virulent in the presence of C2-AuNPs and C2-ZnONPs.

## 3. Discussion

For instance, the biofilm formed by the microbial pathogens represents a key barrier in the failure of antimicrobial therapy [22]. Furthermore, these biofilm-forming bacterial pathogens produce hyper-mutative cells and persister cells, which are more persistent and have more resistance mechanisms than normal cells [9,23]. Hence, the infection issue associated with biofilm-forming bacterial pathogens always encourages the researcher to develop alternative strategies to combat the infection. With the several advantages of the application of nanoparticles in treating infectious and non-infectious diseases [11,24], we aimed to synthesize AuNPs and ZnONPs using biologically derived extracts, particularly from probiotic bacteria. 

Since probiotic bacteria are widely accepted and have been used to colonize the intestines of both humans and animals for a variety of health benefits [25]. Several LAB were isolated from Korean Kimchi in this investigation (collected from Jeju). Among the 10 isolates, isolate C2 was chosen for further characterization due to its high antioxidant activity. C2 was identified as *Leuconostoc* sp. strain C2 and was selected for the synthesis and physiochemical characterization of metal and metal–oxide nanoparticles.

The supernatant from isolate C2 was prepared and employed in the green synthesis of AuNPs and ZnONPs. Several instrumental approaches were used to characterize the produced AuNPs and ZnONPs in detail. Previous work corroborates the preliminary indication of the formation of C2-AuNPs and C2-ZnONPs using the change in color from yellow to dark red wine [26]. Furthermore, increasing the UV–visible absorption peak at 540 nm throughout the reaction was used to confirm the formation of AuNPs and was found to be in close agreement with the prior studies [27,28]. Although no color changes were seen during the synthesis of C2-ZnONPs, the formation of a white precipitate implies the production of nanoparticles, as previously described [26]. The formation of C2-ZnONPs is also indicated by an increase in the UV–vis absorption peak at 330 nm, which is corroborated by prior work [26].

The shape, size, and zeta potential of C2-AuNPs were spherical, 47.77 ± 5.7 nm, and −19.35 ± 0.67 mV, respectively. Previous reports showed that the AuNPs prepared from different bacterial species, such as *Streptomyces* sp. strain NH21 and *Rhodopseudomonas capsulata* have also been reported to be spherical [28,29]. Although the size of AuNPs prepared from the different bacterial species was varied, the size of C2-AuNPs falls in those reported size ranges [28]. The zeta potential of C2-AuNPs demonstrates marginally stable NPs, with values close to previous reports [30]. Similarly, the shape, size, and net charge of C2-ZnONPs were determined to be a rod, 173.77 ± 14.53 nm and 26.62 ± 0.35 mV, respectively. Similar positive zeta potential values have been reported for ZnONPs synthesized employing bacteria such as *Pseudomonas hibiscicola* and *Alkalibacillus* sp.W7 [31,32]. The hexagonal, nanoflower, rod, cube, and spherical shapes of ZnONPs produced using bacterial extract have also been described [33,34]. C2-ZnONPs have a smaller size than ZnONPs-CFS (291.1 nm), which was synthesized using cell-free supernatant from *L. plantarum* TA [33]. The high average zeta potential shows that the synthesized C2-ZnONPs are stable and comparable to previously reported microbially synthesized ZnONPs [19].

According to reports, the conversion of metal ions into metallic nanoparticles requires reducing and capping the agents found in natural extracts [14]. Several metabolites were identified during GC–MS profiling, some of which were functionally described (Supplementary Materials Figure S1 and Table S1). These components have previously been shown to have antioxidant, antimicrobial, and other biological activities [35,36,37]. Based on the GC–MS profiling of the metabolites present in the supernatant, it is inferred that it includes numerous reducing molecules that may be responsible for the formation of nanoparticles. Future research should focus on synthesizing metal and metal–oxide nanoparticles utilizing pure active compounds. C2-AuNPs inhibit the biofilm of *P. aeruginosa*, whereas C2-ZnONPs have antibiofilm efficacy against both *P. aeruginosa* and *S. aureus*. C2-AuNPs and C2-ZnONPs were shown to have a concentration-dependent biofilm inhibitory effect. AuNPs and ZnONPs produced from various bacterial species, as well as pure natural compounds, have been shown to inhibit *P. aeruginosa* and *S. aureus* biofilms in a dose-dependent manner [19]. The maximal biofilm inhibition by C2-ZnONPs against *P. aeruginosa* at 512 µg/mL was equivalent to the previously reported biofilm inhibition by ZnONPs [19,38]. The anti-biofilm effect of these NPs was further validated by employing SEM imaging to examine the biofilm architecture, where cells treated with C2-AuNPs and C2-ZnONPs were either entirely detached or just a few cells adhered to the surface.

Similar studies on the effect of these types of NPs on biofilm architecture have previously been reported [26,39], indicating that the synthesized C2-AuNPs and C2-ZnONPs are potent antibiofilm drugs. The biofilms developed on the surfaces of medical devices and host tissues has become one of the major challenges in the battle against microbial infection [40,41]. The biofilm matrix is extremely thick and composed of extracellular polymeric substances (EPS), and it has been found to function as a barrier to antimicrobial agent entry [42]. The MIC and above MIC of C2-AuNPs and C2-ZnONPs successfully eradicated *P. aeruginosa*, and *S. aureus* developed mature biofilms. The results are consistent with the eradication efficiency of AuNPs and ZnONPs synthesized from various materials against the mature biofilms of *P. aeruginosa* and *S. aureus* [26,43]. Another alternate strategy for controlling pathogenicity and chronic infection caused by *P. aeruginosa* and *S. aureus* has been the diminution of their virulence features [44,45,46]. The sub-MIC of C2-AuNPs and C2-ZnONPs strongly inhibited *P. aeruginosa* motility properties, which is consistent with earlier findings of the nanoparticle-mediated inhibition of virulence features [26,39]. We can decrease the early stage of biofilm formation and the dissemination of bacterial cells throughout the dispersal of a mature biofilm by limiting the motility properties using these NPs [47]. Several additional virulence features of *P. aeruginosa*, such as pyocyanin production, pyoverdine formation, protease activity, and hemolytic activity, were significant at the sub-MIC levels of C2-AuNPs and C2-ZnONPs. The suppression of *P. aeruginosa* virulence properties by C2-AuNPs and C2-ZnONPs was comparable with prior virulence attenuation findings by similar types of nanoparticles [26,39]. The hemolytic activity of *S. aureus* was dose-dependently inhibited by both C2-AuNPs and C2-ZnONPs, which is consistent with prior findings on the inhibitory impact of such NPs. Although the specific mechanism involved in suppressing the biofilm and eradicating the mature biofilm by these nanoparticles is unknown in this study, very little information on the action mechanism of the nanoparticles is available in the literature. The cell membrane breakdown, inhibition of genes associated with biofilm and virulence features, and the formation of cellular reactive oxygen species, which can interfere with cellular metabolism, are the principal effects of NPs on bacterial pathogens [48,49,50,51,52]. To offer clear proof of the mechanism of action of these metal and metal–oxide NPs, more research into the gene expression of genes involved in biofilm development and virulence is necessary.

## 4. Materials and Methods

### 4.1. Microbial Pathogens, Culture Media, and Growth Conditions 

*Escherichia coli* (KCTC1682), *Pseudomonas aeruginosa* PAO1 (KCTC1637), *Listeria monocytogenes* (KCTC3569), and *Staphylococcus aureus* (KCTC 1916) were purchased from the Korean Collection for Type Cultures (KCTC, Daejeon, Korea). *Klebsiella pneumoniae* (ATCC4352) was purchased from American Type Culture Collection (ATCC). *Streptococcus mutans* (KCCM 40105) and *Candida albicans* (KCCM11282) were purchased from the Korean Culture Center of Microorganisms (KCCM; Seodaemun-gu, Seoul, South Korea). Tryptic soy broth (TSB; Difco Laboratory Inc., Detroit, MI, USA) was used to grow *P. aeruginosa*, *S. aureus*, *E. coli*, *L. monocytogenes*, and *Vibrio vulnificus*. For culturing *C. albicans*, the potato dextrose broth (PDB) containing glucose (5 %) was used, whereas for culturing the *S. mutans*, the brain heart infusion (BHI) broth (Difco Laboratory Inc., Detroit, MI, USA) was used. A deMan Rogosa Sharpe Medium (MRS; Difco Laboratory Inc., Detroit, MI, USA) was used to cultivate LAB. Gold (III) chloride trihydrate (CAS# 16961-25-4; purity 99%) and zinc acetate (Zn(CH_3_-COO)_2_·2H_2_O) (CAS # 5970-45-6) were obtained from Sigma-Aldrich Co. (St. Louis, MO, USA). The temperature for the growth of all microbes was 37 °C.

### 4.2. Isolation and Identification of LAB Strains 

The LAB strain was isolated from the Kimchi sample using the previously described method with a small modification [53]. In brief, the 25 g Kimchi sample was diluted 10 times with 0.1 M phosphate-buffered saline (PBS; pH 7.2), and then became homogenous before being employed as an experiment sample. The homogenous sample was inoculated to the surface of MRS agar containing 0.002% (*w/v*) Bromocresol purple (BCP; Duksan Pure Chemical Co., Ltd., Ansan-si, Korea). It was cultured at 37 °C for 24 h–48 h. A single colony was isolated from the agar plate based on the change in yellow color caused by the formation of organic acid by LAB. As described earlier, the isolated LAB strain was taxonomically identified using 16S rRNA gene sequencing [53,54]. The genomic DNA of the LAB isolates was extracted using the extraction Kit (Bioneer, Daejeon, Korea). The universal primer used for sequencing the 16S rRNA gene was 27F (5′-AGAGTTTGATCCTGGCTCAG-3′) and 1492R (5′-GTTACCTGTTACGACTT-3′). The sequencing of the 16S rRNA gene was carried out by the company Bionics (Seoul, Korea). The 16S rRNA gene sequence of strain C2 and a closely related type strain (retrieved from the EzBiOCloud 16S database) were aligned using the multiple sequence alignment program ClustalX version 2.0.12. The phylogenetic tree was built with the neighbor-joining algorithm and the distance estimation method in MEGA 11 [17].

### 4.3. Preparation of LAB Cell-Free Culture Supernatant (CFCS)

LAB strains were cultivated in an MRS medium for 24 h at 37 °C to reach a cell growth value of ~9 log CFU/mL. The supernatant from the growing cell culture was separated by centrifugation (10,000× *g* for 20 min) at 4 °C. The supernatant was filtered via a filter membrane with a pore size of 0.20 µm (DISMIC 25AS020AN, Advantec Toyo, Tokyo, Japan). The resulting supernatant was utilized to test the antibacterial activity and nanoparticle synthesis. In addition, the LAB supernatant was lyophilized using a freeze-dryer (FD8518, ilShinBiobase Co. Ltd., Yangju-si, Korea), and the dried powder was employed for metabolomics analysis using GC–MS.

### 4.4. Synthesis of LAB CFCS-AuNPs and ZnONPs

The previously described technique was used to synthesize C2-AuNPs and C2-ZnONPs [26]. Prior to synthesis, the pH of the CFCS was adjusted to 8.5 using 0.1 M NaOH. To synthesize C2-AuNPs, 1 mM HAuCl_4_∙3H_2_O was dissolved in 200 mL of deionized water and constantly agitated at 70 °C. The prepared CFCS (3 mL) was added dropwise into the heated AuNPs solution and stirred for 2 h at 60 °C. The synthesis of the C2-AuNPs was confirmed by the visual observation of the color changing from yellow to resembling dark red wine. To synthesize C2-ZnONPs, 20 mm Zn(CH_3_-COO)_2_∙2H_2_O was dissolved in deionized water (200 mL) and stirred at 70 °C. The CFCS (3 mL) was added dropwise into the heated solution. After 1 h of continuous stirring at 70 °C, a solution of NaOH (0.1 N) was added dropwise to the solution and left to stir for 2 h. The appearance of a white precipitate indicates the production of C2-ZnONPs. Aside from the visual inspection of the color change, another method used was to scan the UV–vis absorption spectra of the produced NPs from 200 to 800 nm using a microplate reader (BioTek, Winooski, VT, USA). The entire C2-AuNPs solution was first frozen for 30 min at −70 °C before being freeze-dried. On the other hand, the C2-ZnONPs solution was centrifuged (13,000 rpm; 30 min) at 4 °C. Before freeze-drying, the white pellet of C2-ZnONPs was washed and frozen at −70 °C.

### 4.5. Metabolomic Analysis of LAB-Supernatant by GC–MS 

The GC–MS analysis of LAB CFCS was carried out with changes to previous reports [55]. LAB CFCS freeze-dried powder was dissolved in methanol. The presence of metabolites in the samples was determined using gas chromatography–mass spectrometry (GC–MS, Agilent Technologies 7890A-5975C) using helium as the carrier gas at a flow rate of 1.0 mL/min. One microliter of the sample was injected with a 1 min split flow delay and resolved on a 30 m × 0.25 mm × 0.25 µm DB-5MS column (Agilent Technologies, Palo Alto, CA, USA). Temperatures at the inlet, interface, and ion source were 250, 250, and 230 °C, respectively. The oven’s beginning and end temperatures were set at 50 and 230 °C, respectively, at a rate of 5 °C/min for 36 min and then for 2 min at a constant temperature. At 70 eV, electron impact mass spectra from *m*/*z* 50 to 550 were observed. Metabolite identification was accomplished by comparing the mass spectra to analytical standards in the NIST14 database (National Institute of Standards and Technology, Gaithersburg, MD, USA).

### 4.6. Physiochemical Characterization of C2-AuNPs and C2-ZnONPs

In addition to developing dark red wine-colored (C2-AuNPs) and white precipitates (C2-ZnONPs) as preliminary signs, the synthesized NPs were validated by measuring typical UV–vis absorption spectra from 200 to 800 nm. In addition, as previously stated, various other instrumental analyses of the NPs were conducted [26]. Fourier transform infrared spectroscopy (FTIR, JASCO (FT-4100), Tokyo, Japan) was utilized to characterize C2-AuNPs and C2-ZnONPs at frequencies ranging from 4000 to 400 cm^−1^. The morphological shape of the C2-AuNPs and C2-ZnONPs was evaluated using field emission transmission electron microscopy (FE-TEM; JEM-F200, JEOL, Tokyo, Japan). Similarly, the sizes of these NPs were assessed using dynamic light scattering (DLS) and a particle analyzer Litesizer 500 (Anton Paar, GmbH, Graz, Austria). The zeta potentials of C2-AuNPs and C2-ZnONPs were also measured using the particle analyzer Litesizer 500. The energy dispersive X-ray spectroscopy (EDS) and mapping of the metal present in the synthesized C2-AuNPs and C2-ZnONPs were also determined using the FE-TEM instrument, which was also employed to evaluate the morphology. The crystalline nature of the nanoparticles was examined using an X-ray diffractometer (XRD; X-ray diffractometer, Rigaku (Tokyo, Japan), Ultima IV). These completely characterized NPs were employed in antibacterial, antibiofilm, and antivirulence studies against microbial pathogens.

### 4.7. Antibacterial Effects of LAB-Supernatant and Nanoparticles 

The supernatant obtained from the LAB isolate was tested for antimicrobial activity against different microbial pathogens (given in Table 1), as previously described [56]. Furthermore, *Candida albicans* was utilized as a fungal pathogen to test the antifungal efficacy of the LAB supernatant. These microbial pathogens’ seed cultures were cultivated in their respective growth conditions at 37 °C overnight. The culture with an OD_600_ value of 0.05 was put on a 96-well microtiter plate containing CFCS at dilutions ranging from 10^−1^ to 10^−6^. Similarly, the antimicrobial activity of C2-AuNPs and C2-ZnONPs was tested by incubating cell cultures (OD_600 nm_ = 0.05) on a 96-well microtiter plate containing varied nanoparticle concentrations (ranging from 64 to 2048 µg/mL) [26]. The microbial cell culture in the microtiter plate was incubated for 24 h at 37 °C. The OD_600_ value of microbe cell growth was checked using a microtiter plate reader. The OD_600_ value from the test sample (treated with NPs) was subtracted from the OD_600_ value obtained from the control group (containing NPs in growth media without bacterial cells).

### 4.8. Biofilm Inhibition and Eradication of Established Mature Biofilm

The inhibitory effects of C2-AuNPs and C2-ZnONPs on biofilms of *S. aureus* and *P. aeruginosa* were checked the same way as the previously reported method [26]. As a surface for the biofilm formation in the presence of NPs, 96-well microplates were used. The cell culture (OD_600 nm_ = 0.05) of *P. aeruginosa* and *S. aureus* were put on microplates with NPs (ranging from 64 to 1024 µg/mL). Microplates containing cell culture and various doses of NP were incubated at 37 °C for 24 h. The planktonic cells were discarded entirely, and the remaining biofilm cells on the surface were washed thrice using distilled water. Biofilm cells were stained for 20 min with 0.1% crystal violet. These stained cells were again washed three times with distilled water and air-dried. After dissolving the stained cells with 95% alcohol, quantification was carried out at 570 nm. The eradication of the established mature biofilm was accomplished as described earlier [26]. We first established a biofilm in a microplate by adding cell culture (OD_600 nm_ = 0.05) and incubating for 24 h at 37 °C. The planktonic cells were removed aseptically, and the adherent cells were rinsed using sterile TSB. The adhered cells were treated with different concentrations of NPs (ranging from 256 to 2048 µg/mL) and further incubated overnight at 37 °C. The growth media containing free-floating cells from each well was removed, and the stained biofilm cells were quantified at OD at 570 nm. Experiments on initial-stage biofilm inhibition and mature biofilm eradication were carried out in triplicate.

### 4.9. Examination of Biofilms Cells Using SEM

The nanoparticle-affected biofilm cells were examined using advanced microscopy, namely scanning electron microscopy (SEM). The sample that was incubated with nanoparticles was prepared using the methodology described before [26]. In brief, following overnight growth, *P. aeruginosa* and *S. aureus* cell cultures were diluted in fresh TSB to an OD_600_ value of 0.05. These cell cultures were put in a 24-well microplate that had already been fitted with a 0.5 cm × 0.5 cm nylon membrane. C2-AuNPs and C2-ZnONPs at a concentration of 512 µg/mL were used to treat these cell cultures. As a control group, a well-containing nylon membrane was left untreated. After 24 h, the cells were immediately fixed with formaldehyde and glutaraldehyde, then incubated overnight at 4 °C. Unfixed and floating bacterial cells were thrown out, while the cells adhered to the surface were rinsed three times with PBS (pH 7.4). These surface-attached cells were then subjected to varying amounts of ethyl alcohol (50–100%). Following dehydration, these biofilm cells were freeze-dried. Finally, the SEM TESCAN (Vega II LSU), Brno, Czech Republic) was used to investigate the profile of biofilm cells.

### 4.10. Assays for the Inhibition of Multiple Virulence Properties

The quorum sensing (QS)-mediated virulence features of *P. aeruginosa*, such as pyocyanin, pyoverdine, hemolytic, and as well as the protease and hemolytic activity of *S. aureus* in the presence and absence of NPs were determined [57]. The pyocyanin production experiment was started by incubating *P. aeruginosa* cell culture (OD_600 nm_ = 0.05) with varying concentrations (512–64 µg/mL) of C2-AuNPs and C2-ZnONPs. After 24 h incubation at 37 °C, the pink-colored pyocyanin was extracted with chloroform and 0.1 N HCl, and the OD_520 nm_ was measured [58]. Pyoverdine production was measured in the presence and absence of C2-AuNPs and C2-ZnONPs by growing the cell culture in minimal salt media containing 2% sodium succinate. The cell culture was centrifuged (13,000 rpm; 10 min), and pyoverdine was determined by measuring the OD_405 nm_. The attenuation of *P. aeruginosa* and *S. aureus* hemolytic activity by C2-AuNPs and C2-ZnONPs was accomplished by incubating the cell culture (OD_600 nm_ = 0.05) for 24 h at 37 °C in the same manner as previously described [57]. The NPs treated and untreated cell cultures (50 µL) were inoculated into a tube containing 950 µL sheep red blood cells (RBCs; MBcell Ltd., Seoul, Korea) for 2 h at 37 °C. After incubation, the cell culture was centrifuged, and the obtained supernatant was measured at 534 nm. As previously described, the activity of the protein-digesting enzyme protease was investigated using Bacto agar containing casein (5%) [57]. To check the protease activity, the cell culture (OD_600 nm_ = 0.05) was incubated with varying concentrations (128–1024 µg/mL) of C2-AuNPs and C2-ZnONPs. After centrifugation, the collected supernatant was filter sterilized using a 0.2 µ filter. The cell-free supernatant (30 µL) was placed into the hole formed in the casein agar plate and incubated at 37 °C for 24 h. The cleared zone surrounding the hole was measured and compared to the measured zone obtained from the control group. As previously described, *P. aeruginosa* motilities were assessed in the presence or absence of C2-AuNPs and C2-ZnONPs [57]. Swarming was studied by making an agar plate with 0.5% Bacto agar, 0.4% casamino acid, and 0.4% glucose in Luria Bertani (LB) broth. Similarly, for swimming, 0.3% Bacto agar, 1% tryptone, and 0.25% NaCl agar media were made in LB broth and autoclaved. After autoclaving, the swarming and swimming media, C2-AuNPs, and C2-ZnONPs (512 µg/mL) were added to the agar medium and poured into Petri dishes. The overnight grown seed culture (5 µL) of *P. aeruginosa* was placed in the center of the agar plate and incubated for 24 h at 37 °C. The twitching agar medium was made by autoclaving 1.5% Bacto agar, 30 mM glucose, and 0.2% casamino acid (0.2%) in LB. NPs were added to the agar media after autoclaving. Before pouring the agar media, the cell culture was placed in the center of the Petri dishes using a sterile toothpick and aseptically air dried. These Petri plates were filled with twitching agar media. Each of the agar plates was incubated at 37 °C for 24 h. In the case of the swarming and swimming agar plates containing NPs, the surface area traversed by the cells was assessed and compared to the control plates. The twitching motility of the cells on the surface of the Petri dishes was examined by staining with crystal violet after removing the solid agar media, and the dye-stained surface area of the cells was measured.

### 4.11. Statistical Analysis

GraphPad Prism 7.0 (GraphPad Software Inc., San Diego, CA, USA) was used to plot all graphs. The statistical analysis was carried out using one-way ANOVA. *** *p* < 0.0001, ** *p* < 0.01, and * *p* < 0.05 were considered significant. Experiments were repeated three times with triplicate samples.

## 5. Conclusions

In this work, we isolated LAB strain C2 from Kimchi samples, and the isolate was identified as *Leuconostoc* sp. strain C2 based on 16S rRNA gene sequencing. The green synthesis method was used to synthesize AuNPs and ZnONPs with antibiofilm and antivirulence activities against *P. aeruginosa* and *S. aureus* utilizing LAB extract. The biofilm inhibitory activity of C2-AuNPs was shown to be concentration-dependent against *P. aeruginosa*, but not against *S. aureus*. Interestingly, C2-ZnONPs inhibit biofilm development in both *P. aeruginosa* and *S. aureus*. Aside from inhibiting biofilm, these NPs significantly eradicated pre-existing mature biofilms of *P. aeruginosa* and *S. aureus*, and the inhibition was dose-dependent. C2-AuNPs and C2-ZnONPs significantly reduced *P. aeruginosa* virulence features such as hemolytic activity, protease activity, and pyocyanin and pyoverdine production. In contrast, both C2-AuNPs and C2-ZnONPs have been shown to inhibit *S. aureus* hemolytic activity in a dose-dependent manner. Furthermore, these nanoparticles effectively suppress multiple forms of motility in *P. aeruginosa*, including swarming, swimming, and twitching. C2-ZnONPs more effectively inhibit motility (e.g., swimming and twitching) and pyocyanin and pyoverdine production than C2-AuNPs at the same dose. As a result, C2-AuNPs would be a better choice as an antibiofilm and antivirulence agent against human pathogenic bacteria. The current study concludes that these synthesized LAB-based nanoparticles have potential inhibitory effects on the biofilm and virulence features of bacterial pathogens. As a result, we may employ these nanoparticles as possible therapeutic drugs to control infections caused by biofilm-forming bacterial pathogens. A deeper analysis of the molecular action mechanism by undertaking the gene expression studies of the genes associated with the biofilm and virulence features of these bacterial pathogens is necessary in the future.

## Figures and Tables

**Table 1:** MIC values of C2-supernatant, C2-AuNPs, and C2-ZnONPs against different microbial pathogens.

Sample	PA	SA	SM	CA	EC	VV	KP	LM
C-2 supernatant (%)	ND	25	25	50	25	12.5	25	25
C2-AuNPs (µg/mL)	>2048	1024	512	2048	1024	ND	512	2048
C2-ZnONPs (µg/mL)	>2048	512	ND	256	>2048	>2048	>2048	>2048

ND, not determined. PA, *Pseudomonas aeruginosa*; SA, *Staphylococcus aureus*; SM, *Streptococcus mutans*; CA, *Candida albicans*; EC, *Escherichia coli*; VV, *Vibrio vulnificus*; KP, *Klebsiella pneumoniae*; LM, *Listeria monocytogenes*.

## Data Availability

All data are included in the manuscript or supplementary material.

## Supplementary Materials

The following are available online at https://www.mdpi.com/article/10.3390/antibiotics11111524/s1, Figure S1: GC–MS chromatogram of the supernatant obtained from the cell culture of the *Leuconostoc* sp. strain C2. Table S1: Identification of secondary metabolites from *Leuconostoc* sp. strain C2 supernatant using gas chromatograph–mass spectroscopy (GC–MS). References [36,37,59,60,61,62,63,64,65,66,67,68,69,70,71,72,73,74,75,76,77,78,79,80,81,82,83,84,85,86,87,88,89,90,91,92,93,94,95,96,97,98,99,100,101,102] are cited in the supplementary materials.

## References

[1] De Oliveira D.M.P., Forde B.M., Kidd T.J., Harris P.N.A., Schembri M.A., Beatson S.A., Paterson D.L., Walker M.J. (2020). Antimicrobial Resistance in ESKAPE Pathogens. Clin. Microbiol. Rev..

[2] Laxminarayan R., Duse A., Wattal C., Zaidi A.K., Wertheim H.F., Sumpradit N., Vlieghe E., Hara G.L., Gould I.M., Goossens H. (2013). Antibiotic resistance-the need for global solutions. Lancet Infect. Dis..

[3] (2022). Global burden of bacterial antimicrobial resistance in 2019: A systematic analysis. Lancet.

[4] Rice L.B. (2008). Federal funding for the study of antimicrobial resistance in nosocomial pathogens: No ESKAPE. J. Infect. Dis..

[5] Beatson S.A., Walker M.J. (2014). Microbiology. Tracking antibiotic resistance. Science.

[6] Santajit S., Indrawattana N. (2016). Mechanisms of Antimicrobial Resistance in ESKAPE Pathogens. Biomed Res. Int..

[7] de la Fuente-Núñez C., Reffuveille F., Fernández L., Hancock R.E. (2013). Bacterial biofilm development as a multicellular adaptation: Antibiotic resistance and new therapeutic strategies. Curr. Opin. Microbiol..

[8] Mah T.-F.C., O’Toole G.A. (2001). Mechanisms of biofilm resistance to antimicrobial agents. Trends Microbiol..

[9] Khan F., Pham D.T.N., Tabassum N., Oloketuyi S.F., Kim Y.M. (2020). Treatment strategies targeting persister cell formation in bacterial pathogens. Crit. Rev. Microbiol..

[10] Lin Q., Deslouches B., Montelaro R.C., Di Y.P. (2018). Prevention of ESKAPE pathogen biofilm formation by antimicrobial peptides WLBU2 and LL37. Int. J. Antimicrob. Agents.

[11] Jeong G.J., Khan S., Tabassum N., Khan F., Kim Y.M. (2022). Marine-Bioinspired Nanoparticles as Potential Drugs for Multiple Biological Roles. Mar. Drugs.

[12] Cheng Z., Li M., Dey R., Chen Y. (2021). Nanomaterials for cancer therapy: Current progress and perspectives. J. Hematol. Oncol..

[13] Pandit C., Roy A., Ghotekar S., Khusro A., Islam M.N., Emran T.B., Lam S.E., Khandaker M.U., Bradley D.A. (2022). Biological agents for synthesis of nanoparticles and their applications. J. King Saud Univ.-Sci..

[14] Singh J., Dutta T., Kim K.-H., Rawat M., Samddar P., Kumar P. (2018). ‘Green’ synthesis of metals and their oxide nanoparticles: Applications for environmental remediation. J. Nanobiotechnol..

[15] Khan F., Jeong G.-J., Singh P., Tabassum N., Mijakovic I., Kim Y.-M. (2022). Retrospective analysis of the key molecules involved in the green synthesis of nanoparticles. Nanoscale.

[16] Khan I., Bai Y., Zha L., Ullah N., Ullah H., Shah S.R.H., Sun H., Zhang C. (2021). Mechanism of the Gut Microbiota Colonization Resistance and Enteric Pathogen Infection. Front. Cell. Infect. Microbiol..

[17] Tamura K., Stecher G., Kumar S. (2021). MEGA11: Molecular Evolutionary Genetics Analysis Version 11. Mol. Biol. Evol..

[18] Liu Y., Kim S., Kim Y.J., Perumalsamy H., Lee S., Hwang E., Yi T.H. (2019). Green synthesis of gold nanoparticles using *Euphrasia officinalis* leaf extract to inhibit lipopolysaccharide-induced inflammation through NF-κB and JAK/STAT pathways in RAW 264.7 macrophages. Int. J. Nanomed..

[19] Barani M., Masoudi M., Mashreghi M., Makhdoumi A., Eshghi H. (2021). Cell-free extract assisted synthesis of ZnO nanoparticles using aquatic bacterial strains: Biological activities and toxicological evaluation. Int. J. Pharm..

[20] Qin S., Xiao W., Zhou C., Pu Q., Deng X., Lan L., Liang H., Song X., Wu M. (2022). *Pseudomonas aeruginosa*: Pathogenesis, virulence factors, antibiotic resistance, interaction with host, technology advances and emerging therapeutics. Signal Transduct. Target. Ther..

[21] Cheung G.Y.C., Bae J.S., Otto M. (2021). Pathogenicity and virulence of *Staphylococcus aureus*. Virulence.

[22] Stewart P.S. (2002). Mechanisms of antibiotic resistance in bacterial biofilms. Int. J. Med. Microbiol..

[23] Driffield K., Miller K., Bostock J.M., O’Neill A.J., Chopra I. (2008). Increased mutability of *Pseudomonas aeruginosa* in biofilms. J. Antimicrob. Chemother..

[24] Kirtane A.R., Verma M., Karandikar P., Furin J., Langer R., Traverso G. (2021). Nanotechnology approaches for global infectious diseases. Nat. Nanotechnol..

[25] Kechagia M., Basoulis D., Konstantopoulou S., Dimitriadi D., Gyftopoulou K., Skarmoutsou N., Fakiri E.M. (2013). Health benefits of probiotics: A review. ISRN Nutr..

[26] Khan F., Kang M.G., Jo D.M., Chandika P., Jung W.K., Kang H.W., Kim Y.M. (2021). Phloroglucinol-Gold and -Zinc Oxide Nanoparticles: Antibiofilm and Antivirulence Activities towards *Pseudomonas aeruginosa* PAO1. Mar. Drugs.

[27] Singh M., Kalaivani R., Manikandan S., Sangeetha N., Kumaraguru A.K. (2013). Facile green synthesis of variable metallic gold nanoparticle using *Padina gymnospora*, a brown marine macroalga. Appl. Nanosci..

[28] He S., Guo Z., Zhang Y., Zhang S., Wang J., Gu N. (2007). Biosynthesis of gold nanoparticles using the bacteria *Rhodopseudomonas capsulata*. Mater. Lett..

[29] Składanowski M., Wypij M., Laskowski D., Golińska P., Dahm H., Rai M. (2017). Silver and gold nanoparticles synthesized from *Streptomyces* sp. isolated from acid forest soil with special reference to its antibacterial activity against pathogens. J. Clust. Sci..

[30] Li J., Li Q., Ma X., Tian B., Li T., Yu J., Dai S., Weng Y., Hua Y. (2016). Biosynthesis of gold nanoparticles by the extreme bacterium *Deinococcus radiodurans* and an evaluation of their antibacterial properties. Int. J. Nanomed..

[31] Al-Kordy H.M.H., Sabry S.A., Mabrouk M.E.M. (2021). Statistical optimization of experimental parameters for extracellular synthesis of zinc oxide nanoparticles by a novel haloalaliphilic *Alkalibacillus* sp.W7. Sci. Rep..

[32] Punjabi K., Mehta S., Chavan R., Chitalia V., Deogharkar D., Deshpande S. (2018). Efficiency of Biosynthesized Silver and Zinc Nanoparticles Against Multi-Drug Resistant Pathogens. Front. Microbiol..

[33] Mohd Yusof H., Abdul Rahman N.A., Mohamad R., Zaidan U.H., Samsudin A.A. (2020). Biosynthesis of zinc oxide nanoparticles by cell-biomass and supernatant of *Lactobacillus plantarum* TA4 and its antibacterial and biocompatibility properties. Sci. Rep..

[34] Jain D., Shivani, Bhojiya A.A., Singh H., Daima H.K., Singh M., Mohanty S.R., Stephen B.J., Singh A. (2020). Microbial Fabrication of Zinc Oxide Nanoparticles and Evaluation of Their Antimicrobial and Photocatalytic Properties. Front. Chem..

[35] Ziklo N., Bibi M., Salama P. (2021). The Antimicrobial Mode of Action of Maltol and Its Synergistic Efficacy with Selected Cationic Surfactants. Cosmetics.

[36] Guo L., Wang A., Sun Y., Xu C. (2012). Evaluation of antioxidant and immunity function of tetramethylpyrazine phosphate tablets in vivo. Molecules.

[37] Edeeva S.E., Kopylova G.N., Bakaeva Z.V., Samonina G.E., Umarova B.A., Guseva A.A. (2008). Protective and therapeutic effects of glyprolines in psychoemotional stress induced by cholecystokinin-4 injection. Bull. Exp. Biol. Med..

[38] Abinaya M., Vaseeharan B., Divya M., Sharmili A., Govindarajan M., Alharbi N.S., Kadaikunnan S., Khaled J.M., Benelli G. (2018). Bacterial exopolysaccharide (EPS)-coated ZnO nanoparticles showed high antibiofilm activity and larvicidal toxicity against malaria and Zika virus vectors. J. Trace Elem. Med. Biol..

[39] Khan F., Manivasagan P., Lee J.W., Pham D.T.N., Oh J., Kim Y.M. (2019). Fucoidan-Stabilized Gold Nanoparticle-Mediated Biofilm Inhibition, Attenuation of Virulence and Motility Properties in *Pseudomonas aeruginosa* PAO1. Mar. Drugs.

[40] Khatoon Z., McTiernan C.D., Suuronen E.J., Mah T.F., Alarcon E.I. (2018). Bacterial biofilm formation on implantable devices and approaches to its treatment and prevention. Heliyon.

[41] Dongari-Bagtzoglou A. (2008). Pathogenesis of mucosal biofilm infections: Challenges and progress. Expert Rev. Anti Infect..

[42] Flemming H.-C., Wingender J. (2010). The biofilm matrix. Nat. Rev. Microbiol..

[43] Husain F.M., Qais F.A., Ahmad I., Hakeem M.J., Baig M.H., Masood Khan J., Al-Shabib N.A. (2022). Biosynthesized Zinc Oxide Nanoparticles Disrupt Established Biofilms of Pathogenic Bacteria. Appl. Sci..

[44] Hentzer M., Wu H., Andersen J.B., Riedel K., Rasmussen T.B., Bagge N., Kumar N., Schembri M.A., Song Z., Kristoffersen P. (2003). Attenuation of *Pseudomonas aeruginosa* virulence by quorum sensing inhibitors. EMBO J..

[45] Lee J.-H., Cho H.S., Kim Y., Kim J.-A., Banskota S., Cho M.H., Lee J. (2013). Indole and 7-benzyloxyindole attenuate the virulence of *Staphylococcus aureus*. Appl. Microbiol. Biotechnol..

[46] Salam A.M., Quave C.L. (2018). Targeting Virulence in *Staphylococcus aureus* by Chemical Inhibition of the Accessory Gene Regulator System In Vivo. mSphere.

[47] Khan F., Pham D.T.N., Oloketuyi S.F., Kim Y.M. (2020). Regulation and controlling the motility properties of *Pseudomonas aeruginosa*. Appl. Microbiol. Biotechnol..

[48] Lee J.-H., Kim Y.-G., Cho M.H., Lee J. (2014). ZnO nanoparticles inhibit *Pseudomonas aeruginosa* biofilm formation and virulence factor production. Microbiol. Res..

[49] Dwivedi S., Wahab R., Khan F., Mishra Y.K., Musarrat J., Al-Khedhairy A.A. (2014). Reactive oxygen species mediated bacterial biofilm inhibition via zinc oxide nanoparticles and their statistical determination. PLoS ONE.

[50] Kaur T., Putatunda C., Vyas A., Kumar G. (2021). Zinc oxide nanoparticles inhibit bacterial biofilm formation via altering cell membrane permeability. Prep. Biochem. Biotechnol..

[51] Perveen K., Husain F.M., Qais F.A., Khan A., Razak S., Afsar T., Alam P., Almajwal A.M., Abulmeaty M.M.A. (2021). Microwave-Assisted Rapid Green Synthesis of Gold Nanoparticles Using Seed Extract of *Trachyspermum ammi*: ROS Mediated Biofilm Inhibition and Anticancer Activity. Biomolecules.

[52] Lin Y.K., Yang S.C., Hsu C.Y., Sung J.T., Fang J.Y. (2021). The Antibiofilm Nanosystems for Improved Infection Inhibition of Microbes in Skin. Molecules.

[53] Won S.-M., Chen S., Park K.W., Yoon J.-H. (2020). Isolation of lactic acid bacteria from kimchi and screening of *Lactobacillus sakei* ADM14 with anti-adipogenic effect and potential probiotic properties. LWT.

[54] Fazlurrahman, Batra M., Pandey J., Suri C.R., Jain R.K. (2009). Isolation and characterization of an atrazine-degrading *Rhodococcus* sp. strain MB-P1 from contaminated soil. Lett. Appl. Microbiol..

[55] Mao Y., Zhang X., Xu Z. (2020). Identification of antibacterial substances of Lactobacillus plantarum DY-6 for bacteriostatic action. Food Sci. Nutr..

[56] Jo D.-M., Park S.-K., Khan F., Kang M.-G., Lee J.-H., Kim Y.-M. (2021). An approach to extend the shelf life of ribbonfish fillet using lactic acid bacteria cell-free culture supernatant. Food Control.

[57] Khan F., Lee J.W., Pham D.T.N., Lee J.H., Kim H.W., Kim Y.K., Kim Y.M. (2020). Streptomycin mediated biofilm inhibition and suppression of virulence properties in *Pseudomonas aeruginosa* PAO1. Appl. Microbiol. Biotechnol..

[58] Essar D.W., Eberly L., Hadero A., Crawford I.P. (1990). Identification and characterization of genes for a second anthranilate synthase in *Pseudomonas aeruginosa*: Interchangeability of the two anthranilate synthases and evolutionary implications. J. Bacteriol..

[59] Gilardoni G., Clericuzio M., Tosi S., Zanoni G., Vidari G. (2007). Antifungal acylcyclopentenediones from fruiting bodies of *Hygrophorus chrysodon*. J. Nat. Prod..

[60] Mokhtari M., Jackson M.D., Brown A.S., Ackerley D.F., Ritson N.J., Keyzers R.A., Munkacsi A.B. (2018). Bioactivity-Guided Metabolite Profiling of Feijoa (*Acca sellowiana*) Cultivars Identifies 4-Cyclopentene-1,3-dione as a Potent Antifungal Inhibitor of Chitin Synthesis. J. Agric. Food Chem..

[61] Maier N.K., Leppla S.H., Moayeri M. (2015). The cyclopentenone prostaglandin 15d-PGJ2 inhibits the NLRP1 and NLRP3 inflammasomes. J. Immunol..

[62] Martinez A., Alonso M., Castro A., Dorronsoro I., Gelpí J.L., Luque F.J., Pérez C., Moreno F.J. (2005). SAR and 3D-QSAR studies on thiadiazolidinone derivatives: Exploration of structural requirements for glycogen synthase kinase 3 inhibitors. J. Med. Chem..

[63] Lizardi L., Garcia M.C., Sanchez J.A., Zuazaga C. (1992). Sulfhydryl alkylating agents induce calcium current in skeletal muscle fibers of a crustacean (*Atya lanipes*). J. Membr. Biol..

[64] Bui T., Straus D.S. (1998). Effects of cyclopentenone prostaglandins and related compounds on insulin-like growth factor-I and Waf1 gene expression. Biochim. Biophys. Acta.

[65] Uto Y., Nagasawa H., Jin C.Z., Nakayama S., Tanaka A., Kiyoi S., Nakashima H., Shimamura M., Inayama S., Fujiwara T. (2008). Design of antiangiogenic hypoxic cell radiosensitizers: 2-nitroimidazoles containing a 2-aminomethylene-4-cyclopentene-1,3-dione moiety. Bioorg. Med. Chem..

[66] Du L., King J.B., Morrow B.H., Shen J.K., Miller A.N., Cichewicz R.H. (2012). Diarylcyclopentendione metabolite obtained from a *Preussia typharum* isolate procured using an unconventional cultivation approach. J. Nat. Prod..

[67] Neumann P., Brodhun F., Sauer K., Herrfurth C., Hamberg M., Brinkmann J., Scholz J., Dickmanns A., Feussner I., Ficner R. (2012). Crystal structures of *Physcomitrella patens* AOC1 and AOC2: Insights into the enzyme mechanism and differences in substrate specificity. Plant Physiol..

[68] Braga S.F., Alves É.V., Ferreira R.S., Fradico J.R., Lage P.S., Duarte M.C., Ribeiro T.G., Júnior P.A., Romanha A.J., Tonini M.L. (2014). Synthesis and evaluation of the antiparasitic activity of bis-(arylmethylidene) cycloalkanones. Eur. J. Med. Chem..

[69] Beranič N., Stefane B., Brus B., Gobec S., Rižner T.L. (2013). New enzymatic assay for the AKR1C enzymes. Chem. Biol. Interact..

[70] Li J., Zhang D., Wu X. (2011). Synthesis and biological evaluation of novel exo-methylene cyclopentanone tetracyclic diterpenoids as antitumor agents. Bioorg. Med. Chem. Lett..

[71] Yamada K., Shimizu A., Komatsu H., Sakata R., Ohta A. (1994). Effects of 2,5-dimethylpyrazine on plasma testosterone and polyamines- and acid phosphatase-levels in the rat prostate. Biol. Pharm. Bull..

[72] Yamada K., Watanabe Y., Aoyagi Y., Ohta A. (2001). Effect of alkylpyrazine derivatives on the duration of pentobarbital-induced sleep, picrotoxicin-induced convulsion and gamma-aminobutyric acid (GABA) levels in the mouse brain. Biol. Pharm. Bull..

[73] Yamada K., Kobayashi Y., Fujihara H., Ohta A. (1998). Inhibitory effect of 2,5-dimethylpyrazine on oxytocic agent-induced uterine hypercontraction of normal or pregnant female rats. Biol. Pharm. Bull..

[74] Chen Z., Liu Q., Zhao Z., Bai B., Sun Z., Cai L., Fu Y., Ma Y., Wang Q., Xi G. (2021). Effect of hydroxyl on antioxidant properties of 2,3-dihydro-3,5-dihydroxy-6-methyl-4H-pyran-4-one to scavenge free radicals. RSC Adv..

[75] Yu X., Zhao M., Liu F., Zeng S., Hu J. (2013). Identification of 2,3-dihydro-3,5-dihydroxy-6-methyl-4H-pyran-4-one as a strong antioxidant in glucose–histidine Maillard reaction products. Food Res. Int..

[76] Čechovská L., Cejpek K., Konečný M., Velíšek J. (2011). On the role of 2,3-dihydro-3,5-dihydroxy-6-methyl-(4H)-pyran-4-one in antioxidant capacity of prunes. Eur. Food Res. Technol..

[77] Suganuma H., Inakuma T., Kikuchi Y. (2002). Amelioratory Effect of Barley Tea Drinking on Blood Fluidity. J. Nutr. Sci. Vitaminol..

[78] Yanagimoto K., Lee K.G., Ochi H., Shibamoto T. (2002). Antioxidative activity of heterocyclic compounds found in coffee volatiles produced by Maillard reaction. J. Agric. Food Chem..

[79] Hidalgo F.J., Nogales F., Zamora R. (2003). Effect of the Pyrrole Polymerization Mechanism on the Antioxidative Activity of Nonenzymatic Browning Reactions. J. Agric. Food Chem..

[80] Devadas S.M., Nayak U.Y., Narayan R., Hande M.H., Ballal M. (2019). 2,5-Dimethyl-4-hydroxy-3(2H)-furanone as an Anti-biofilm Agent Against Non-*Candida albicans Candida* Species. Mycopathologia.

[81] Sung W.S., Jung H.J., Park K., Kim H.S., Lee I.-S., Lee D.G. (2007). 2,5-dimethyl-4-hydroxy-3(2H)-furanone (DMHF); antimicrobial compound with cell cycle arrest in nosocomial pathogens. Life Sci..

[82] Chuang P.-H., Lee C.-W., Chou J.-Y., Murugan M., Shieh B.-J., Chen H.-M. (2007). Anti-fungal activity of crude extracts and essential oil of *Moringa oleifera* Lam. Bioresour. Technol..

[83] Wang J.-q., Zhang L., Tao X.-g., Wei L., Liu B., Huang L.-l., Chen Y.-g. (2013). Tetramethylpyrazine upregulates the aquaporin 8 expression of hepatocellular mitochondria in septic rats. J. Surg. Res..

[84] Sun Y., Jiang J., Zhang Z., Yu P., Wang L., Xu C., Liu W., Wang Y. (2008). Antioxidative and thrombolytic TMP nitrone for treatment of ischemic stroke. Bioorganic Med. Chem..

[85] Sun Y., Song M., Niu L., Bai X., Sun N., Zhao X., Jiang J., He J., Li H. (2013). Antiviral effects of the constituents derived from Chinese herb medicines on infectious bursal disease virus. Pharm. Biol..

[86] Ren Z., Ma J., Zhang P., Luo A., Zhang S., Kong L., Qian C. (2012). The effect of ligustrazine on L-type calcium current, calcium transient and contractility in rabbit ventricular myocytes. J. Ethnopharmacol..

[87] Wang H., Jenner A.M., Lee C.-Y.J., Shui G., Tang S.Y., Whiteman M., Wenk M.R., Halliwell B. (2007). The identification of antioxidants in dark soy sauce. Free Radic. Res..

[88] Chaves S., Canário S., Carrasco M.P., Mira L., Santos M.A. (2012). Hydroxy(thio)pyrone and hydroxy(thio)pyridinone iron chelators: Physico-chemical properties and anti-oxidant activity. J. Inorg. Biochem..

[89] Yang M.-L., Kuo P.-C., Hwang T.-L., Wu T.-S. (2011). Anti-inflammatory Principles from *Cordyceps sinensis*. J. Nat. Prod..

[90] Kandioller W., Hartinger C.G., Nazarov A.A., Bartel C., Skocic M., Jakupec M.A., Arion V.B., Keppler B.K. (2009). Maltol-Derived Ruthenium–Cymene Complexes with Tumor Inhibiting Properties: The Impact of Ligand–Metal Bond Stability on Anticancer Activity In Vitro. Chem.-Eur. J..

[91] Amatori S., Ambrosi G., Fanelli M., Formica M., Fusi V., Giorgi L., Macedi E., Micheloni M., Paoli P., Pontellini R. (2012). Synthesis, Basicity, Structural Characterization, and Biochemical Properties of Two [(3-Hydroxy-4-pyron-2-yl)methyl]amine Derivatives Showing Antineoplastic Features. J. Org. Chem..

[92] Watanabe-Akanuma M., Inaba Y., Ohta T. (2007). Mutagenicity of UV-irradiated maltol in *Salmonella typhimurium*. Mutagenesis.

[93] Kang K.S., Tanaka T., Cho E.J., Yokozawa T. (2009). Evaluation of the Peroxynitrite Scavenging Activity of Heat-Processed Ginseng. J. Med. Food.

[94] Lalitha N., Sadashivaiah B., Ramaprasad T.R., Singh S.A. (2020). Anti-hyperglycemic activity of myricetin, through inhibition of DPP-4 and enhanced GLP-1 levels, is attenuated by co-ingestion with lectin-rich protein. PLoS ONE.

[95] Ichimura T., Yamanaka A., Otsuka T., Yamashita E., Maruyama S. (2009). Antihypertensive Effect of Enzymatic Hydrolysate of Collagen and Gly-Pro in Spontaneously Hypertensive Rats. Biosci. Biotechnol. Biochem..

[96] Abdurraafi M., Dermawan A., Julianti E., Putra M., Karim F. (2019). Identification and Evaluation of Antibacterial Compounds from the Vibrio sp. associated with the Ascidian Pycnoclavella diminuta. Pharm. Sci. Res..

[97] Lee S., Eom S., Nguyen K.V.A., Lee J., Park Y., Yeom H.D., Lee J.H. (2022). The Application of the Neuroprotective and Potential Antioxidant Effect of Ergotamine Mediated by Targeting N-Methyl-D-Aspartate Receptors. Antioxidants.

[98] Vesely D.L. (1983). Ergotamine and dihydroergotamine enhance guanylate cyclase activity. Res. Commun. Chem. Pathol. Pharm..

[99] Kruk-Slomka M., Michalak A., Biala G. (2015). Antidepressant-like effects of the cannabinoid receptor ligands in the forced swimming test in mice: Mechanism of action and possible interactions with cholinergic system. Behav. Brain Res..

[100] Nojima H., Ohba Y., Kita Y. (2007). Oleamide derivatives are prototypical anti-metastasis drugs that act by inhibiting Connexin 26. Curr. Drug Saf..

[101] Lechin F., Van Der Dijs B., Bentolila A., Peña F. (1977). Antidiarrheal effects of dihydroergotamine. J. Clin. Pharm..

[102] Silberstein S.D. (1997). The pharmacology of ergotamine and dihydroergotamine. Headache.

