# Supplementary material for: Antibiofilm and Antivirulence Activities of Gold and Zinc Oxide Nanoparticles Synthesized from Kimchi-Isolated Leuconostoc sp. Strain C2
Source: Antibiotics (Basel). 2022 Nov 1;11(11):1524. doi: 10.3390/antibiotics11111524 (PMC9686622; doi:10.3390/antibiotics11111524)
Supplement: Supplementary file 1 [file antibiotics-11-01524-s001.zip › antibiotics-2009106-supplementary.pdf]

Supporting Information:

Title: Antibiofilm and antivirulence activities of gold and zinc oxide nanoparticles synthesized from Kimchi-isolated *Leuconostoc* sp. strain C2

Min-Gyun Kang[1], Fazlurrahman Khan[2,3*], Du-Min Jo[1,2,3], DoKyung Oh[1,2,3], Nazia Tabassum[2,3] and Young-Mog Kim[1,2,3*]

1 Department of Food Science and Technology, Pukyong National University, Busan 48513, Republic of Korea.
2 Marine Integrated Biomedical Technology Center, The National Key Research Institutes in Universities, Pukyong National University, Busan 48513, Republic of Korea
3 Research Center for Marine Integrated Bionics Technology, Pukyong National University, Busan 48513, Republic of Korea

***** Correspondence: Fazlurrahman Khan and Young-Mog Kim
Phone: +82-51-629-5832; Fax: +82-51-629-5824
E-mail: fkhan055@pknu.ac.kr and ymkim@pknu.ac.kr

Figure legends:

Figure S1. GC-MS chromatogram of the supernatant obtained from the cell culture of the *Leuconostoc* sp. strain C2.

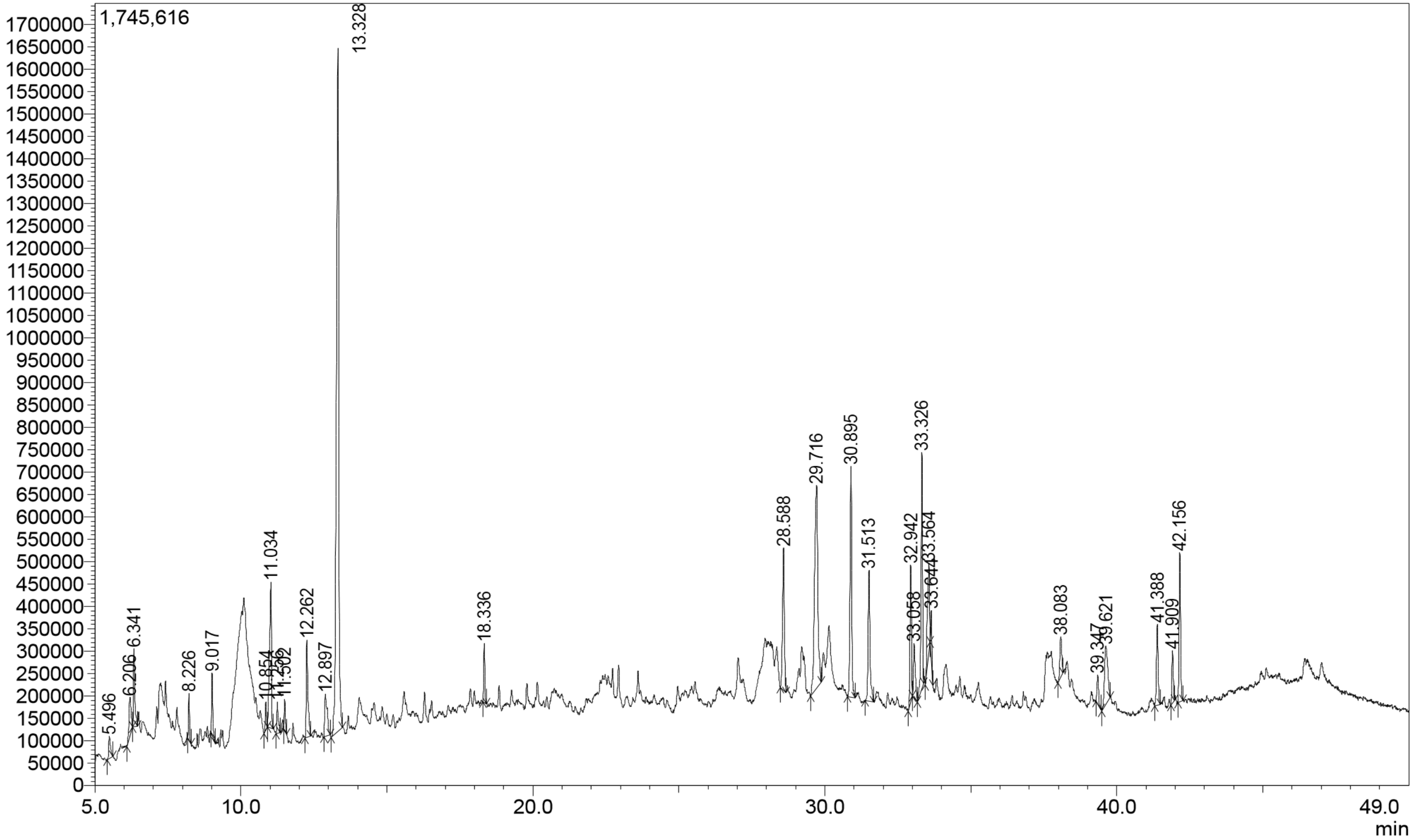

Peak Report TIC

Table S1. Identification of secondary metabolites from *Leuconostoc* sp. strain C2 supernatant using Gas Chromatograph-Mass Spectroscopy

Name	R.Time	Composition(%)	SI	Chemical structure	Biological acitivity	Reference
Protoanemonine<br>4-Cyclopentene-1,3-dione<br>1,3-Cyclopentenedione<br>2-cyclopentene-1,4-dione<br>CYCLOPENT-2-EN-1,4-DIONE	5.495	0.73	90<br>88<br>88<br>87<br>85	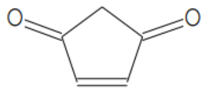	• Antifungal activity<br>• Anti-inflammatory<br>• Glycogen synthase kinase inhibitors<br>• Increase in Ca^{2+} influx<br>• Regulation of growth-related and stress-induced genes<br>• Antiangiogenic activity	[1-7]
CYCLOPENTANONE<br>Adipic ketone	6.205	1.43	85<br>85	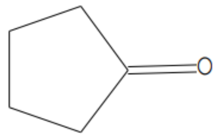	• Antioxidant activity	[3,8-12]

Dumasin<br>Ketocyclopentane<br><br>Dihydropyran			85<br>85		• Regulation of oxylipins<br>• Anti-inflammatory<br>• Antiparasitic activity<br>• Antitumor agents<br>• Regulation of cancers	
Pyrazine, 2,5-dimethyl-<br><br>2,5-Dimethylpyrazine<br><br>Pyrazine, 2,6-dimethyl-	6.34	1.78	97<br><br>97<br><br>96	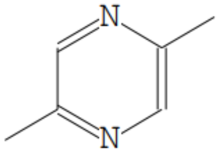	• Effects of testosterone<br>• Strengthen the GABAnergic system<br>• Inhibit oxytocin- and prostaglandin	[13-15]
2,4-Dihydroxy-2,5-dimethyl-3(2H)-furan-3-one	8.225	0.78	94	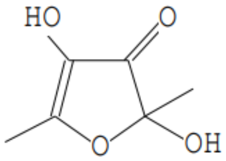	• Antioxidant activity	[16-18]

2,3-Dihydro-3,5-dihydroxy-6-methyl-4H-pyran-4-one			85			
4H-Pyran-4-one, 2,3-dihydro-3,5-dihydroxy-6-methyl- (CAS)			82			
Trimethylpyrazine	9.015	1.17		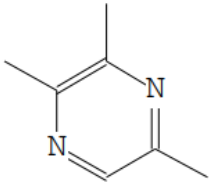	• Effect of blood fluidity	[19]
Pyrazine, trimethyl- (CAS)			95			
Ethanone, 1-(1H-pyrrol-2-yl)- (CAS)	10.855	0.51	96	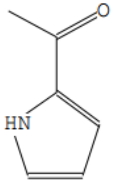	• Antioxidant activity	[20,21]
2-Acetylpyrrole			95			
2,5-Dimethyl-4-hydroxy-3(2H)-furanone	11.035	4.8	95	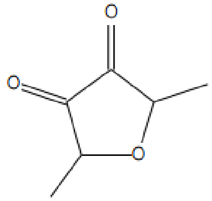	• Antibiofilm activity<br>• Antimicrobial activity	[22,23]
3-FURANONE, 2,3-DIHYDRO-4-HYDROXY-2,5-DIMETHYL-			95			
Pyrazine, 3-ethyl-2,5-dimethyl- (CAS)	11.255	0.69	93	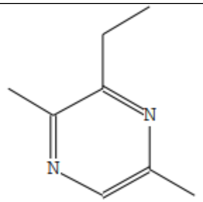	• Anti-fungal activity	[24]
2-Ethyl-3,6-dimethylpyrazine			90			
3,5-Dimethyl-2-ethylpyrazine			90			

Pyrazine, tetramethyl-	11.5	0.58	96	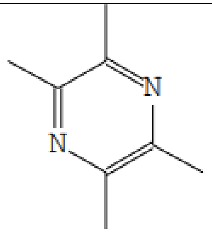	• Effect of reducing myocardial damage<br>• Effect of protecting hepatocellular mitochondria<br>• Antioxidant activity<br>• Antiviral effects<br>• Effect of ligustrazine	[25-29]
Tetramethylpyrazine			95			
2,3,5,6-Tetramethylpyrazine $$ tetramethyl pyrazine			94			
4H-PYRAN-4-ONE, 3-HYDROXY-2-METHYL-	12.26	2.75	95	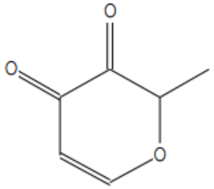	• Antioxidant activity<br>• Anti-inflammatory<br>• Anticancer activity<br>• Antineoplastic	[30-36]
Larixic acid			94			
Veltol			93			
Maltol			92			

					• Mutagenicity<br>• Peroxynitrite scavenging activity	
4H-Pyran-4-one, 2,3-dihydro-3,5-dihydroxy-6-methyl- (CAS)	13.33	27.89	92	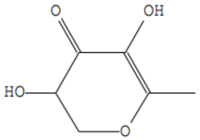	• Antioxidant activity	[18]
3,5-DIHYDROXY-2-METHYL-5,6-DIHYDROPYRAN-4-ONE			92			
Pyrazine, 2,5-dimethyl-3-(3-methylbutyl)-	18.335	1.14	94	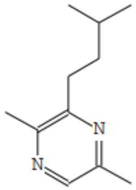	-	-
2,5-DIMETHYL-3-(3-METHYLBUTYL-)PYRAZINE			94			
3-Isoamyl-2,5-dimethylpyrazine			94			
5-Isopentyl-2,3-dimethylpyrazine			93			
Pyrrolo[1,2-a]pyrazine-1,4-dione, hexahydro-	29.715	11.23	97	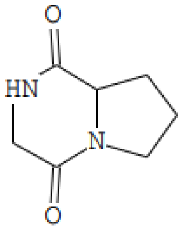	• Protective and therapeutic effects of glyprolines<br>• Anti-hypergly	[37-39]
1,4-diaza-2,5-dioxobicyclo[4.3.0]nonane			95			
Glycyl-L-proline			90			

1,4-Diazabicyclo[4.3.0]nonan-2,5-dione, N-acetyl-			84		cemic activity<br>● Antihypertensive effect	
3-Buten-2-one, 4-(1-aziridinyl)-4-(dimethylamino)- (CAS)			83			
Pyrrolo[1,2-a]pyrazine-1,4-dione, hexahydro-3-(2-methylpropyl)-	32.94	3.14	92	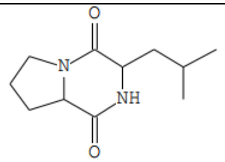	● Antibacterial activity	[40]
1,4-diaza-2,5-dioxo-3-isobutyl bicyclo[4.3.0]nonane			91			
L-Proline, N-valeryl-, heptadecyl ester			81			
l-Leucine, N-cyclopropylcarbonyl-, pentadecyl ester			80			
Pyrrolo[1,2-a]pyrazine-1,4-dione, hexahydro-3-(2-methylpropyl)-	33.325	6.09	96	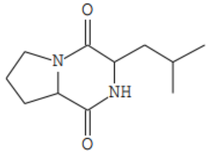	● Antibacterial activity	[40]
1,4-diaza-2,5-dioxo-3-isobutyl bicyclo[4.3.0]nonane			95			
cyclo-(Pro-Leu)			80			
L-Proline, N-valeryl-, heptadecyl ester			80			
L-Proline, N-valeryl-, octadecyl ester			79			
Pyrrolo[1,2-a]pyrazine-1,4-dione, hexahydro-3-(2-methylpropyl)-	33.565	3.16	92	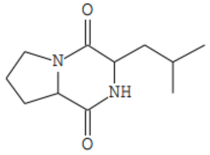	● Antibacterial activity	[40]
1,4-diaza-2,5-dioxo-3-isobutyl bicyclo[4.3.0]nonane			91			
L-Proline, N-valeryl-, tetradecyl ester			79			

L-Proline, N-valeryl-, pentadecyl ester			79			
Pyrrolo[1,2-a]pyrazine-1,4-dione, hexahydro-3-(phenylmethyl)-	41.39	2.38	92	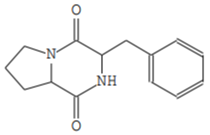	• Antioxidant activity<br>• Enhancing guanylate cyclase activity.	[41,42]
3-benzyl-1,4-diaza-2,5-dioxobicyclo[4.3.0]nonane			91			
Dihydroergotamine			91			
ERGOTAMINE			90			
9-Octadecenamide (CAS)	41.91	1.19	90	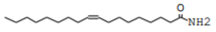	• Antidepressant-like effects<br>• Anti-metastasis drugs	[43,44]
Armid ow			90			
Adogen 73			89			
Oleamide			87			
Pyrrolo[1,2-a]pyrazine-1,4-dione, hexahydro-3-(phenylmethyl)-	42.155	3.81	92	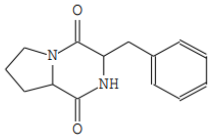	• Antidiarrheal activity<br>• Antagonist activity<br>• Enhancing guanylate cyclase activity.	[42,45,46]
3-benzyl-1,4-diaza-2,5-dioxobicyclo[4.3.0]nonane			92			
Dihydroergotamine			87			
DIHYDROERGOCRISTINE			81			

References

1. Gilardoni, G.; Clericuzio, M.; Tosi, S.; Zanoni, G.; Vidari, G. Antifungal acylcyclopentenediones from fruiting bodies of Hygrophorus chrysodon. *J Nat Prod* **2007**, *70*, 137-139, doi:10.1021/np060512c.
2. Mokhtari, M.; Jackson, M.D.; Brown, A.S.; Ackerley, D.F.; Ritson, N.J.; Keyzers, R.A.; Munkacsi, A.B. Bioactivity-Guided Metabolite Profiling of Feijoa (Acca sellowiana) Cultivars Identifies 4-Cyclopentene-1,3-dione as a Potent Antifungal Inhibitor of Chitin Synthesis. *J Agric Food Chem* **2018**, *66*, 5531-5539, doi:10.1021/acs.jafc.7b06154.
3. Maier, N.K.; Leppla, S.H.; Moayeri, M. The cyclopentenone prostaglandin 15d-PGJ2 inhibits the NLRP1 and NLRP3 inflammasomes. *J Immunol* **2015**, *194*, 2776-2785, doi:10.4049/jimmunol.1401611.
4. Martinez, A.; Alonso, M.; Castro, A.; Dorronsoro, I.; Gelpí, J.L.; Luque, F.J.; Pérez, C.; Moreno, F.J. SAR and 3D-QSAR studies on thiadiazolidinone derivatives: exploration of structural requirements for glycogen synthase kinase 3 inhibitors. *J Med Chem* **2005**, *48*, 7103-7112, doi:10.1021/jm040895g.
5. Lizardi, L.; Garcia, M.C.; Sanchez, J.A.; Zuazaga, C. Sulfhydryl alkylating agents induce calcium current in skeletal muscle fibers of a crustacean (Atya lanipes). *J Membr Biol* **1992**, *129*, 167-178, doi:10.1007/bf00219512.
6. Bui, T.; Straus, D.S. Effects of cyclopentenone prostaglandins and related compounds on insulin-like growth factor-I and Waf1 gene expression. *Biochim Biophys Acta* **1998**, *1397*, 31-42, doi:10.1016/s0167-4781(97)00214-5.
7. Uto, Y.; Nagasawa, H.; Jin, C.Z.; Nakayama, S.; Tanaka, A.; Kiyoi, S.; Nakashima, H.; Shimamura, M.; Inayama, S.; Fujiwara, T.; et al. Design of antiangiogenic hypoxic cell radiosensitizers: 2-nitroimidazoles containing a 2-aminomethylene-4-cyclopentene-1,3-dione moiety. *Bioorg Med Chem* **2008**, *16*, 6042-6053, doi:10.1016/j.bmc.2008.04.041.
8. Du, L.; King, J.B.; Morrow, B.H.; Shen, J.K.; Miller, A.N.; Cichewicz, R.H. Diarylcyclopentendione metabolite obtained from a Preussia typharum isolate procured using an unconventional cultivation approach. *J Nat Prod* **2012**, *75*, 1819-1823, doi:10.1021/np300473h.
9. Neumann, P.; Brodhun, F.; Sauer, K.; Herrfurth, C.; Hamberg, M.; Brinkmann, J.; Scholz, J.; Dickmanns, A.; Feussner, I.; Ficner, R. Crystal structures of Physcomitrella patens AOC1 and AOC2: insights into the enzyme mechanism and differences in substrate specificity. *Plant Physiol* **2012**, *160*, 1251-1266, doi:10.1104/pp.112.205138.
10. Braga, S.F.; Alves É, V.; Ferreira, R.S.; Fradico, J.R.; Lage, P.S.; Duarte, M.C.; Ribeiro, T.G.; Júnior, P.A.; Romanha, A.J.; Tonini, M.L.; et al. Synthesis and evaluation of the antiparasitic activity of bis-(arylmethylidene) cycloalkanones. *Eur J Med Chem* **2014**, *71*, 282-289, doi:10.1016/j.ejmech.2013.11.011.
11. Beranič, N.; Stefane, B.; Brus, B.; Gobec, S.; Rižner, T.L. New enzymatic assay for the AKR1C enzymes. *Chem Biol Interact* **2013**, *202*, 204-209, doi:10.1016/j.cbi.2012.12.003.
12. Li, J.; Zhang, D.; Wu, X. Synthesis and biological evaluation of novel exo-methylene

cyclopentanone tetracyclic diterpenoids as antitumor agents. *Bioorg Med Chem Lett* **2011**, *21*, 130-132, doi:10.1016/j.bmcl.2010.11.055.

13. Yamada, K.; Shimizu, A.; Komatsu, H.; Sakata, R.; Ohta, A. Effects of 2,5-dimethylpyrazine on plasma testosterone and polyamines- and acid phosphatase-levels in the rat prostate. *Biol Pharm Bull* **1994**, *17*, 730-731, doi:10.1248/bpb.17.730.
14. Yamada, K.; Watanabe, Y.; Aoyagi, Y.; Ohta, A. Effect of alkylpyrazine derivatives on the duration of pentobarbital-induced sleep, picrotoxicin-induced convulsion and gamma-aminobutyric acid (GABA) levels in the mouse brain. *Biol Pharm Bull* **2001**, *24*, 1068-1071, doi:10.1248/bpb.24.1068.
15. Yamada, K.; Kobayashi, Y.; Fujihara, H.; Ohta, A. Inhibitory effect of 2,5-dimethylpyrazine on oxytocic agent-induced uterine hypercontraction of normal or pregnant female rats. *Biol Pharm Bull* **1998**, *21*, 538-540, doi:10.1248/bpb.21.538.
16. Chen, Z.; Liu, Q.; Zhao, Z.; Bai, B.; Sun, Z.; Cai, L.; Fu, Y.; Ma, Y.; Wang, Q.; Xi, G. Effect of hydroxyl on antioxidant properties of 2,3-dihydro-3,5-dihydroxy-6-methyl-4H-pyran-4-one to scavenge free radicals. *RSC Advances* **2021**, *11*, 34456-34461, doi:10.1039/D1RA06317K.
17. Yu, X.; Zhao, M.; Liu, F.; Zeng, S.; Hu, J. Identification of 2,3-dihydro-3,5-dihydroxy-6-methyl-4H-pyran-4-one as a strong antioxidant in glucose–histidine Maillard reaction products. *Food Research International* **2013**, *51*, 397-403, doi:https://doi.org/10.1016/j.foodres.2012.12.044.
18. Čechovská, L.; Cejpek, K.; Konečný, M.; Velíšek, J. On the role of 2,3-dihydro-3,5-dihydroxy-6-methyl-(4H)-pyran-4-one in antioxidant capacity of prunes. *European Food Research and Technology* **2011**, *233*, 367-376, doi:10.1007/s00217-011-1527-4.
19. Suganuma, H.; Inakuma, T.; Kikuchi, Y. Amelioratory Effect of Barley Tea Drinking on Blood Fluidity. *Journal of Nutritional Science and Vitaminology* **2002**, *48*, 165-168, doi:10.3177/jnsv.48.165.
20. Yanagimoto, K.; Lee, K.G.; Ochi, H.; Shibamoto, T. Antioxidative activity of heterocyclic compounds found in coffee volatiles produced by Maillard reaction. *J Agric Food Chem* **2002**, *50*, 5480-5484, doi:10.1021/jf025616h.
21. Hidalgo, F.J.; Nogales, F.; Zamora, R. Effect of the Pyrrole Polymerization Mechanism on the Antioxidative Activity of Nonenzymatic Browning Reactions. *Journal of Agricultural and Food Chemistry* **2003**, *51*, 5703-5708, doi:10.1021/jf034369u.
22. Devadas, S.M.; Nayak, U.Y.; Narayan, R.; Hande, M.H.; Ballal, M. 2,5-Dimethyl-4-hydroxy-3(2H)-furanone as an Anti-biofilm Agent Against Non-Candida albicans Candida Species. *Mycopathologia* **2019**, *184*, 403-411, doi:10.1007/s11046-019-00341-y.
23. Sung, W.S.; Jung, H.J.; Park, K.; Kim, H.S.; Lee, I.-S.; Lee, D.G. 2,5-dimethyl-4-hydroxy-3(2H)-furanone (DMHF); antimicrobial compound with cell cycle arrest in nosocomial pathogens. *Life Sciences* **2007**, *80*, 586-591, doi:https://doi.org/10.1016/j.lfs.2006.10.008.
24. Chuang, P.-H.; Lee, C.-W.; Chou, J.-Y.; Murugan, M.; Shieh, B.-J.; Chen, H.-M. Anti-fungal activity of crude extracts and essential oil of Moringa oleifera Lam. *Bioresource Technology*

2007, *98*, 232-236, doi:https://doi.org/10.1016/j.biortech.2005.11.003.

25. Guo, L.; Wang, A.; Sun, Y.; Xu, C. Evaluation of Antioxidant and Immunity Function of Tetramethylpyrazine Phosphate Tablets in Vivo. *Molecules* **2012**, *17*, 5412-5421.
26. Wang, J.-q.; Zhang, L.; Tao, X.-g.; Wei, L.; Liu, B.; Huang, L.-l.; Chen, Y.-g. Tetramethylpyrazine upregulates the aquaporin 8 expression of hepatocellular mitochondria in septic rats. *Journal of Surgical Research* **2013**, *185*, 286-293, doi:https://doi.org/10.1016/j.jss.2013.05.106.
27. Sun, Y.; Jiang, J.; Zhang, Z.; Yu, P.; Wang, L.; Xu, C.; Liu, W.; Wang, Y. Antioxidative and thrombolytic TMP nitrone for treatment of ischemic stroke. *Bioorganic & Medicinal Chemistry* **2008**, *16*, 8868-8874, doi:https://doi.org/10.1016/j.bmc.2008.08.075.
28. Sun, Y.; Song, M.; Niu, L.; Bai, X.; Sun, N.; Zhao, X.; Jiang, J.; He, J.; Li, H. Antiviral effects of the constituents derived from Chinese herb medicines on infectious bursal disease virus. *Pharm Biol* **2013**, *51*, 1137-1143, doi:10.3109/13880209.2013.781197.
29. Ren, Z.; Ma, J.; Zhang, P.; Luo, A.; Zhang, S.; Kong, L.; Qian, C. The effect of ligustrazine on L-type calcium current, calcium transient and contractility in rabbit ventricular myocytes. *Journal of Ethnopharmacology* **2012**, *144*, 555-561, doi:https://doi.org/10.1016/j.jep.2012.09.037.
30. Wang, H.; Jenner, A.M.; Lee, C.-Y.J.; Shui, G.; Tang, S.Y.; Whiteman, M.; Wenk, M.R.; Halliwell, B. The identification of antioxidants in dark soy sauce. *Free Radical Research* **2007**, *41*, 479-488, doi:10.1080/10715760601110871.
31. Chaves, S.; Canário, S.; Carrasco,M.P.; Mira, L.; Santos, M.A. Hydroxy(thio)pyrone and hydroxy(thio)pyridinone iron chelators: Physico-chemical properties and anti-oxidant activity. *Journal of Inorganic Biochemistry* **2012**, *114*, 38-46, doi:https://doi.org/10.1016/j.jinorgbio.2012.04.019.
32. Yang, M.-L.; Kuo, P.-C.; Hwang, T.-L.; Wu, T.-S. Anti-inflammatory Principles from Cordyceps sinensis. *Journal of Natural Products* **2011**, *74*, 1996-2000, doi:10.1021/np100902f.
33. Kandioller, W.; Hartinger, C.G.; Nazarov, A.A.; Bartel, C.; Skocic, M.; Jakupec, M.A.; Arion, V.B.; Keppler, B.K. Maltol-Derived Ruthenium–Cymene Complexes with Tumor Inhibiting Properties: The Impact of Ligand–Metal Bond Stability on Anticancer Activity In Vitro. *Chemistry – A European Journal* **2009**, *15*, 12283-12291, doi:https://doi.org/10.1002/chem.200901939.
34. Amatori, S.; Ambrosi, G.; Fanelli, M.; Formica, M.; Fusi, V.; Giorgi, L.; Macedi, E.; Micheloni, M.; Paoli, P.; Pontellini, R.; et al. Synthesis, Basicity, Structural Characterization, and Biochemical Properties of Two [(3-Hydroxy-4-pyron-2-yl)methyl]amine Derivatives Showing Antineoplastic Features. *The Journal of Organic Chemistry* **2012**, *77*, 2207-2218, doi:10.1021/jo202270j.
35. Watanabe-Akanuma, M.; Inaba, Y.; Ohta, T. Mutagenicity of UV-irradiated maltol in Salmonella typhimurium. *Mutagenesis* **2007**, *22*, 43-47, doi:10.1093/mutage/gel057.
36. Kang, K.S.; Tanaka, T.; Cho, E.J.; Yokozawa, T. Evaluation of the Peroxynitrite Scavenging Activity of Heat-Processed Ginseng. *Journal of Medicinal Food* **2009**, *12*, 124-130,

doi:10.1089/jmf.2007.0646.

37. Edeeva, S.E.; Kopylova, G.N.; Bakaeva, Z.V.; Samonina, G.E.; Umarova, B.A.; Guseva, A.A. Protective and therapeutic effects of glyprolines in psychoemotional stress induced by cholecystokinin-4 injection. *Bulletin of Experimental Biology and Medicine* **2008**, *145*, 302-306, doi:10.1007/s10517-008-0076-8.
38. Lalitha, N.; Sadashivaiah, B.; Ramaprasad, T.R.; Singh, S.A. Anti-hyperglycemic activity of myricetin, through inhibition of DPP-4 and enhanced GLP-1 levels, is attenuated by co-ingestion with lectin-rich protein. *PLoS One* **2020**, *15*, e0231543, doi:10.1371/journal.pone.0231543.
39. Ichimura, T.; Yamanaka, A.; Otsuka, T.; Yamashita, E.; Maruyama, S. Antihypertensive Effect of Enzymatic Hydrolysate of Collagen and Gly-Pro in Spontaneously Hypertensive Rats. *Bioscience, Biotechnology, and Biochemistry* **2009**, *73*, 2317-2319, doi:10.1271/bbb.90197.
40. Abdurraafi, M.; Dermawan, A.; Julianti, E.; Putra, M.; Karim, F. Identification and Evaluation of Antibacterial Compounds from the Vibrio sp. associated with the Ascidian Pycnoclavella diminuta. *Pharmaceutical Sciences and Research* **2019**, *6*.
41. Lee, S.; Eom, S.; Nguyen, K.V.A.; Lee, J.; Park, Y.; Yeom, H.D.; Lee, J.H. The Application of the Neuroprotective and Potential Antioxidant Effect of Ergotamine Mediated by Targeting N-Methyl-D-Aspartate Receptors. *Antioxidants (Basel)* **2022**, *11*, doi:10.3390/antiox11081471.
42. Vesely, D.L. Ergotamine and dihydroergotamine enhance guanylate cyclase activity. *Res Commun Chem Pathol Pharmacol* **1983**, *40*, 245-254.
43. Kruk-Slomka, M.; Michalak, A.; Biala, G. Antidepressant-like effects of the cannabinoid receptor ligands in the forced swimming test in mice: mechanism of action and possible interactions with cholinergic system. *Behav Brain Res* **2015**, *284*, 24-36, doi:10.1016/j.bbr.2015.01.051.
44. Nojima, H.; Ohba, Y.; Kita, Y. Oleamide derivatives are prototypical anti-metastasis drugs that act by inhibiting Connexin 26. *Curr Drug Saf* **2007**, *2*, 204-211, doi:10.2174/157488607781668837.
45. Lechin, F.; Van Der Dijs, B.; Bentolila, A.; Peña, F. Antidiarrheal effects of dihydroergotamine. *J Clin Pharmacol* **1977**, *17*, 339-349, doi:10.1002/j.1552-4604.1977.tb04614.x.
46. Silberstein, S.D. The pharmacology of ergotamine and dihydroergotamine. *Headache* **1997**, *37 Suppl 1*, S15-25.
